# AXL Promotes Ischemic Myelin Repair Through Alleviating Myelin Debris Deposition and Lipid Droplets Accumulation

**DOI:** 10.1002/advs.202517825

**Published:** 2026-01-12

**Authors:** Junqiu Jia, Yonghui Gan, Jia Li, Lilin Li, Hailan Meng, Min Sun, Lei Ye, Rui Mao, Xiang Cao, Shengnan Xia, Xinyu Bao, Renyuan Liu, Meijuan Zhang, Yun Xu

**Affiliations:** ^1^ Department of Neurology Nanjing Drum Tower Hospital Chinese Academy of Medical Science & Peking Union Medical College Nanjing China; ^2^ Department of Emergency Medicine Nanjing Drum Tower Hospital Nanjing Drum Tower Hospital Clinical College of Nanjing University of Chinese Medicine Nanjing China; ^3^ Department of Neurology Nanjing Drum Tower Hospital Affiliated Hospital of Medical School Nanjing University Nanjing China; ^4^ State Key Laboratory of Pharmaceutical Biotechnology and Institute of Translational Medicine for Brain Critical Diseases Nanjing University Nanjing China; ^5^ Jiangsu Key Laboratory for Molecular Medicine and Institute of Translational Medicine for Brain Critical Diseases Nanjing University Nanjing China; ^6^ Jiangsu Provincial Key Discipline of Neurology Nanjing China; ^7^ Nanjing Neurology Clinical Medical Center and Nanjing Gulou Hospital Brain disease and brain Science Center Nanjing China; ^8^ Department of Radiology Nanjing Drum Tower Hospital Affiliated Hospital of Medical School Nanjing University Nanjing China

**Keywords:** AXL, ischemic stroke, lipid droplet, microglia, myelin debris

## Abstract

Ischemic white matter injury leads to long‐term neurological deficits but currently lacks effective therapies. Although AXL has been implicated in debris clearance and inflammatory regulation, its role in post‐stroke myelin repair remains unclear. Here, we report robust upregulation of microglial AXL in mice after tMCAO. Microglial AXL cKO mice exhibited worse motor and cognitive deficits up to 28 days after tMCAO, accompanied by more severe white matter damage, increased myelin debris, and greater lipid droplets (LDs) accumulation in microglia than WT controls. Longitudinal analysis showed that AXL‐deficient microglia had reduced early phagocytic capacity but increased LDs accumulation and lipid peroxidation at later stages. Transcriptomic profiling revealed altered inflammatory and sphingolipid metabolism pathways in AXL‐deficient microglia. Mechanistically, AXL regulates *Smpd1* transcription via EGR1, thereby modulating sphingolipid metabolism and LDs accumulation. Remarkably, supplement with ASM (the *Smpd1*‐encoded enzyme) in AXL cKO mice reduced LDs accumulation and attenuated ischemic white matter injury. Collectively, these findings identify microglial AXL as an endogenous regulator of myelin repair after ischemic stroke.

## Graphical abstract

Microglial AXL drives white matter repair after stroke by orchestrating the cleanup of myelin debris. Mechanistically, AXL signals through EGR1 to boost *Smpd1* transcription, regulating sphingolipid metabolism and preventing lipid droplet toxicity. Restoring the pathway with ASM therapy mitigates damage, positioning AXL as a key node for therapeutic intervention.

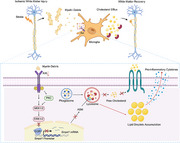

**Abbreviations**
AD: Alzheimer's disease
ASM: sphingomyelinase
cKO: conditional knockout
CSVD: cerebral small vessel diseases
FA: fractional anisotropy
Gas6: growth arrest specific protein 6
LDs: lipid droplets
LDAM: lipid‐droplet accumulating microglia
MD: mean diffusivity
MWM: Morris water maze
MS: multiple sclerosis
OAM: OGD followed by myelin debris stimulation
OGD: oxygen‐glucose deprivation
OPCs: oligodendrocyte precursor cells
*SMPD1*
: Sphingomyelin Phosphodiesterase 1
tMCAO: transient middle cerebral artery occlusion
TREM2: triggering receptor expressed on myeloid cells 2
WMI: white matter injury
WT: wildtype

## 1 Introduction

Ischemic stroke, a leading cause of mortality and disability worldwide, is influenced by the integrity of cerebral white matter, which plays a pivotal role in both disease progression and recovery [1]. White matter injury (WMI) contributes to persistent sensorimotor and cognitive deficits in stroke patients [2]. Clarifying mechanisms of post‐ischemic white matter repair may unveil novel therapeutic targets for neurorehabilitation, with the potential to extend beyond the narrow time window of acute stroke interventions.

Microglia are central to WMI repair after stroke, mediating myelin debris clearance, modulating neuroinflammation, secreting neurotrophic factors, and promoting remyelination [3, 4, 5]. AXL, a TAM (Tyro3, Axl, MerTK) receptor tyrosine kinase expressed on glia, binds endogenous ligands Gas6 and Protein S, with the highest affinity for Gas6 [6]. Structurally, AXL contains extracellular immunoglobulin‐like/fibronectin type III domains and an intracellular kinase with immunoreceptor tyrosine‐based inhibitory motif (ITIM) domains that transduce signals via phosphorylation [7].

TAM‐mediated phagocytosis is increasingly recognized in neurological disease. In Alzheimer's disease (AD), microglia use TAM receptors to detect and clear Aβ plaques; enhancing TAM signaling with a Gas6 fusion protein promotes plaque removal and improves behavior [8, 9]. In aged white matter, microglial AXL is abundant and supports clearance of degenerated myelin [10]. After ischemic stroke, microglial AXL remains upregulated throughout recovery, persisting to 84 days post‐injury [11]. Gas6/AXL signaling is protective in the acute phase of cerebral vascular disease, enhancing microglial clearance of apoptotic neurons or eryptotic erythrocytes and dampening inflammation [12, 13]. However, the role of AXL in post‐stroke myelin repair, especially in late phases, remains unclear.

Beyond phagocytosing lipid‐rich myelin debris, effective white matter repair also critically depends on the dynamic metabolism of internalized lipid [14, 15]. Abundant myelin debris clearance may overwhelm the metabolic capacity of phagocytes, which induce multiple maladaptive biological effects. In demyelinating conditions, overaccumulation of lipids such as cholesterol esters within lipid droplets (LDs) in aged phagocytes can disrupt phagolysosomal integrity, trigger inflammasome activation, and impair remyelination [15]. Microglia with excessive LDs adopt a dysfunctional, pro‐inflammatory state that hinders regeneration [16, 17]. Debris‐activated receptors can couple phagocytosis to lipid homeostasis; for example, TREM2‐deficient microglia maintain uptake but fail in lipid breakdown, leading to pathological LDs accumulation and failed repair [18, 19]. Sphingolipids (key myelin components) help maintain LDs homeostasis [20, 21]; myelin debris and sphingomyelin overload lysosomes and bias microglia toward pro‐inflammatory states [22]. Whether AXL similarly coordinates lipid metabolism after debris engulfment is unknown.

Here, we examine how microglial AXL regulates ischemic myelin repair, focusing on myelin debris clearance and lipid metabolism. Using in vivo and in vitro stroke models, we show that AXL deficiency impairs functional recovery, exacerbates white‐matter injury, increases LDs accumulation, and elevates lipid peroxidation. Mechanistically, AXL modulates sphingolipid metabolism by regulating EGR1‐driven *Smpd1* transcription. These findings identify AXL as a key regulator of post‐stroke microglial function and a promising target to enhance white‐matter repair.

## 2 Results

### 2.1 Microglial AXL Increases Profoundly after Ischemic Stroke in Mice

**FIGURE 1:**
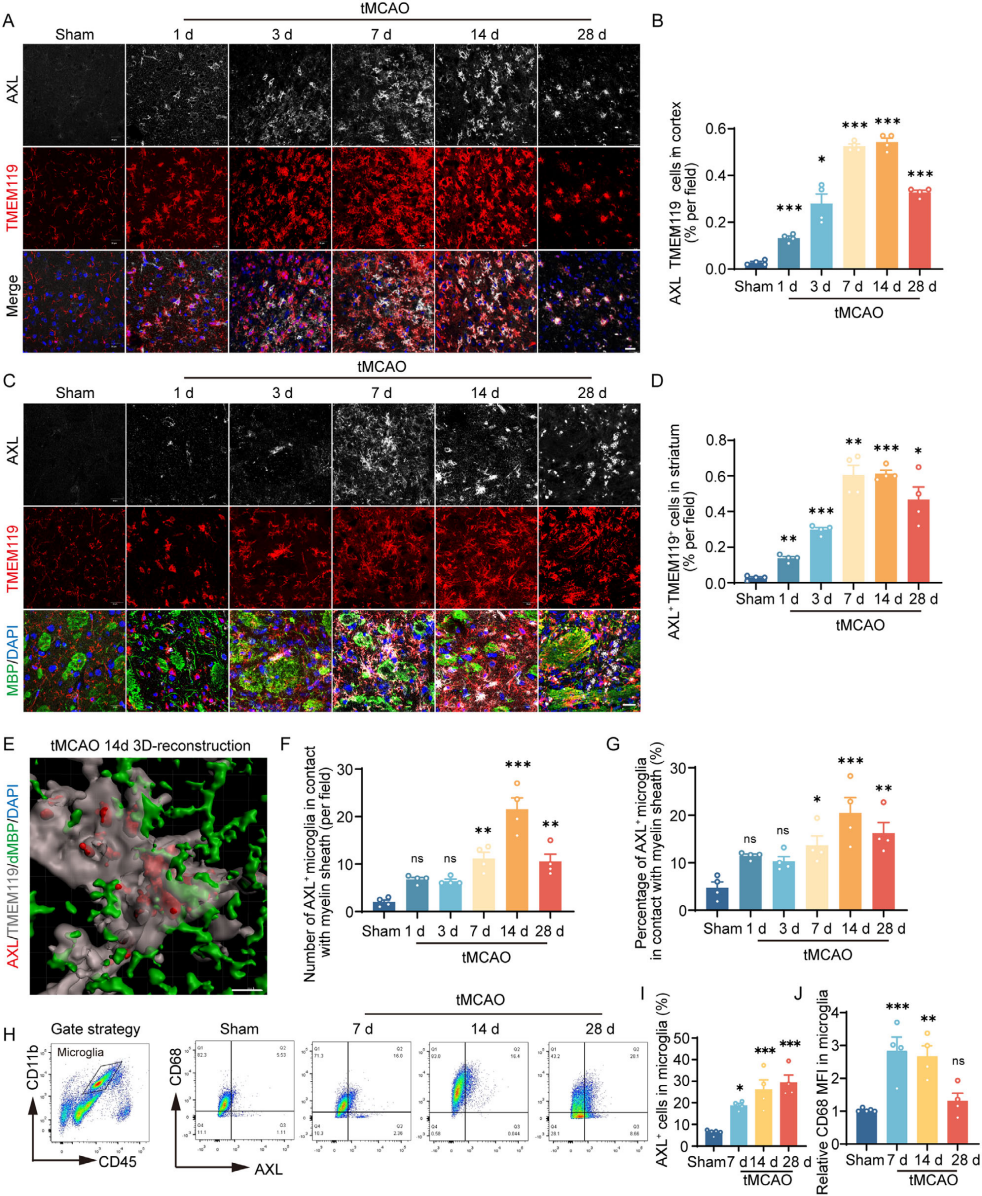
Microglial AXL increases profoundly after ischemic stroke in mice. (A, B) Representative immunofluorescence staining (A) and percentages (B) of AXL^+^TMEM119^+^ cells in the cortex of the ischemic hemisphere in sham group or at different timepoints after tMCAO. Scale bar, 20 µm. *n* = 4 mice per group. (C,D) Representative immunofluorescence staining (C) and percentages (D) of AXL^+^TMEM119^+^ cells in the striatum of the ischemic hemisphere in sham group or at different timepoints after tMCAO. Scale bar, 20 µm. *n* = 4 mice per group. (E) Representative 3D‐reconsrtuction images of AXL^+^ microglia engulfed myelin debris. Scale bar, 5 µm. (F,G) The number per field (F) and the percentage of AXL^+^ microglia (G) in contact with myelin sheath in the striatum of the ischemic hemisphere in sham group or at different timepoints after tMCAO. *n* = 4. (H) Gating strategy of flow cytometry (left). Representative flow cytometric analysis for the expression of AXL and CD68 in CD11b^+^CD45^int^ cells in sham, tMCAO 7d, 14d, and 28d (right). (I) Percentage of AXL^+^ microglia in the ischemic hemisphere in sham group or at 7d, 14d and 28d after tMCAO. *n* ≥ 4 per group. (J) Relative CD68 mean fluorescence intensity in microglia. *n* ≥ 4 per group. Values were mean ± SEM. *p < 0.05, **p < 0.01, and ***p < 0.001.

We mapped the spatial–temporal pattern of AXL by immunofluorescence. Consistent with our microglial transcriptome sorting at serial time points after transient middle cerebral artery occlusion (tMCAO) surgery (Figure S1A) [23], microglial AXL was upregulated in the cortex and striatum, peaking at 7–14 days post‐stroke (Figure 1A–D). AXL‐positive microglia colocalized with myelin sheaths in the striatum and showed active phagocytosis of myelin debris by 3D reconstruction, most prominently at day 14 (Figure 1C–G). Flow cytometry corroborated a continued increase in microglial AXL through day 28 after tMCAO (Figure 1H,I). Consistent with the robust phagocytic activity, the expression of CD68, a lysosomal marker, was elevated in the ischemic hemisphere at days 7 and 14 (Figure 1J). Overall, AXL is robustly upregulated in post‐stroke microglia, which cluster around myelin and phagocytose myelin debris.

### 2.2 Microglial AXL Ameliorates Long‐Term Stroke Outcome

**FIGURE 2:**
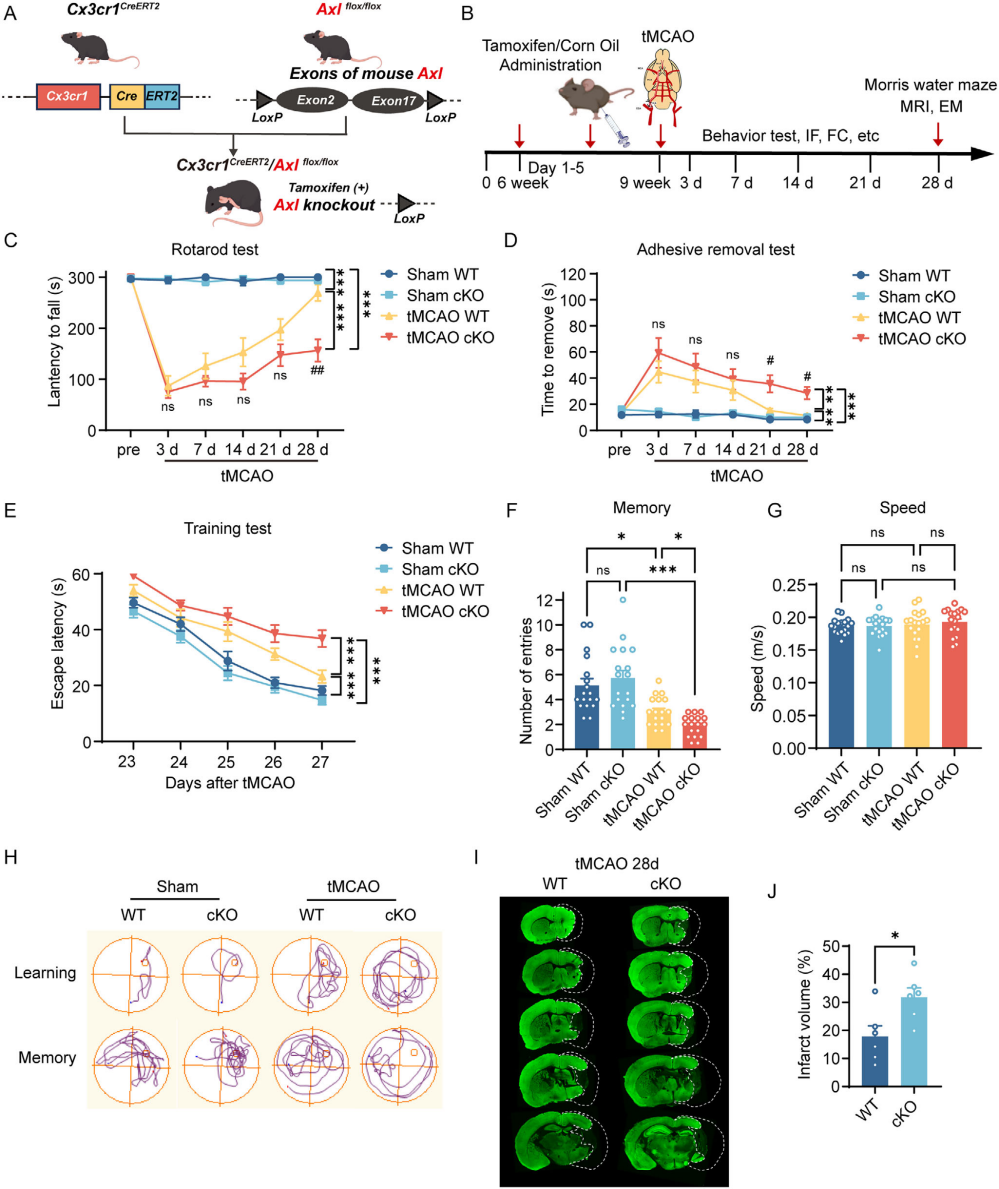
Microglial AXL ameliorates long‐term stroke outcome. (A) Schematic illustration of breeding of microglial conditional knockout *Axl* mice. (B) Overview of the experimental timeline. Tamoxifen administration was initiated 3 weeks before tMCAO surgery. Behavioral tests, immunofluorescence, and flow cytometry were conducted 3, 7, 14, 21, and 28 days after surgery. Morris water maze was performed 23–28 days after surgery. MRI and electron microscopy were performed 28 days after surgery. IF: immunofluorescence. FC: flow cytometry. EM: electron microscopy. (C,D) Motor function and sensory coordination function of mice were evaluated by rotarod test (C) and adhesive removal test (D). *n* = 18 per group. ^#^ *p* < 0.05, ^##^ *p* < 0.01, and ns not significant compared between tMCAO WT and tMCAO cKO group on each day. (E–G) Cognitive functions including training test for learning (E), probe test for memory (F), and speed (G) were evaluated by Morris water maze test. *n* = 18 per group. (H) Representative images showed the swim paths in the Morris water maze. (I,J) Representative images of MAP2 immunofluorescence staining (I) and infarct volume statistics (J) in WT and AXL cKO mice 28 days after tMCAO. Values were mean ± SEM. *p < 0.05, **p < 0.01, ***p < 0.001, and ns not significant.

To define the function of microglial AXL in ischemic stroke, we generated Axl*^flox/flox^* mice and crossed them with *Cx3cr1^CreERT2^* to enable tamoxifen‐inducible deletion in *Cx3cr1* ^+^ cells (AXL cKO); Axl*^flox/flox^* littermates served as WT controls (Figure 2A). Knockout efficiency was validated at protein and mRNA levels (Figure S1B–D). We assessed behavior before and after tMCAO using the rotarod, adhesive removal, and Morris water maze (MWM) tests (Figure 2B). Survival did not differ between groups (Figure S1E). From days 21–28, AXL cKO mice showed worse motor performance (rotarod test) and sensorimotor function (adhesive removal test) than WT (Figure 2C,D). In the MWM, AXL cKO mice had longer escape latencies during training (poorer spatial learning; Figure 2E) and fewer platform‐region entries on probe testing (poorer memory retention; Figure 2F,H), with no difference in swimming speed (Figure 2G). T2‐weighted MRI and MAP2 staining at day 28 revealed larger infarct/atrophy volumes in AXL cKO mice (Figure S1F; Figure 2I,J). These data indicate that microglial AXL supports long‐term neurological recovery after stroke.

### 2.3 Microglial AXL Facilitates Myelin Debris Clearance and Oligodendrocyte Maturation, Which Thereby Promotes Ischemic Myelin Repair

**FIGURE 3:**
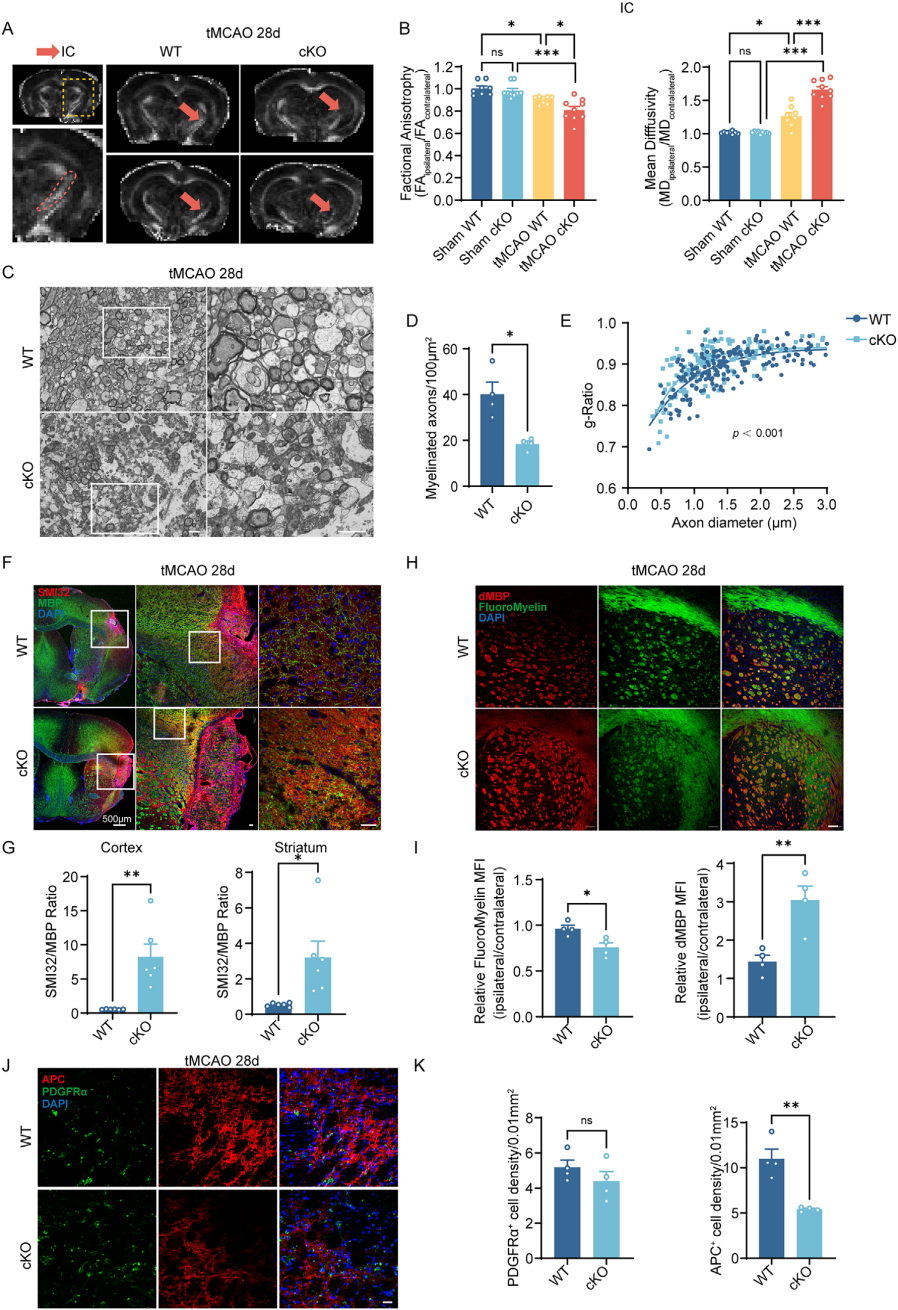
Microglial AXL facilitates myelin debris clearance and oligodendrocyte maturation, which thereby promotes ischemic myelin repair. (A) Representative FA maps of the WT and AXL cKO mouse brain on the 28d post‐tMCAO. Red arrowheads pointed IC. (B) Quantification of FA value (left) and MD value (right) expressed as the ratio of ipsilateral values to the contralateral values in the IC area. *n* ≥ 8 per group. (C) Representative electron micrographs showed myelinated axons in the ipsilateral striatum of WT and AXL cKO ischemic mice at day 28 post‐tMCAO. Scale bar, 2 µm. (D,E) Quantification of myelinated axons (D) and g‐ratio (E) in the ipsilateral striatum of WT and AXL cKO ischemic mice at day 28 post‐tMCAO. *n* = 4 per group, 203 fibers for WT mice and 142 fibers for AXL cKO mice have been analyzed. (F,G) Representative immunofluorescence staining of SMI32 and MBP in the cortex and striatum area (F) and ratios of SMI32 to MBP staining intensity (G) of WT and AXL cKO mice at day 28 post‐tMCAO. Scale bar, 20 µm. *n* = 6 per group. (H,I) Quantification of fluoromyelin MFI (left) and dMBP MFI (right) expressed as the ratio of ipsilateral values to the contralateral values (H) in striatum and the representative immunofluorescence images (I) of WT and AXL cKO mice at day 28 post‐tMCAO. *n* = 4 per group. Scale bar, 20 µm. (J,K) Representative immunofluorescence images and the analysis of APC and PDGFRα in WT and AXL cKO mice at tMCAO 28d. Scale bar, 20 µm. *n* = 4 per group. Values were mean ± SEM. *p < 0.05, **p < 0.01, ***p < 0.001, and ns not significant.

Given the spatial association of AXL^+^ microglia with myelin, we evaluated white‐matter injury at day 28 by MRI. Fractional anisotropy (FA) and mean diffusivity (MD) indicated poorer myelin integrity in cKO mice: the FA ratio (ipsilateral/contralateral) was lower and the MD ratio was higher in the internal capsule (IC) versus WT (Figure 3A,B). Consistently, this imaging‐observed change of white matter has been further validated in the cortical and striatum regions (Figure S1G,H). Electron microscopy showed fewer myelinated axons and lower g‐ratio values in cKO mice (Figure 3C–E). The SMI32/MBP ratio, an additional marker of axonal/myelin integrity, was significantly higher in AXL cKO mice in the cortex and striatum (Figure 3F,G). Using dMBP to label myelin debris and FluoroMyelin to label intact myelin, immunofluorescence demonstrated greater debris deposition in cKO, especially in the striatum (Figure 3H,I). Mechanistically, we further investigate whether AXL affects white matter repair by directly impacting oligodendrocytes. At 28 days post‐tMCAO, immunostaining showed that microglial AXL deficiency did not alter the number of PDGFRα^+^ oligodendrocyte precursor cells (OPCs), while a significant reduction in APC^+^ mature oligodendrocytes was observed in AXL cKO mice (Figure 3J,K). These findings indicate that AXL promotes post‐stroke myelin repair primarily by facilitating the differentiation of OPCs into mature myelinating oligodendrocytes rather than affecting OPCs pool expansion. Together with the behavioral data, these results show that AXL deficiency exacerbates myelin‐debris accumulation and compromises OPC maturation, which thereby impairs myelin integrity at four weeks post‐tMCAO.

### 2.4 Microglial AXL Alleviates Lipid Droplets Deposition after Stroke

**FIGURE 4:**
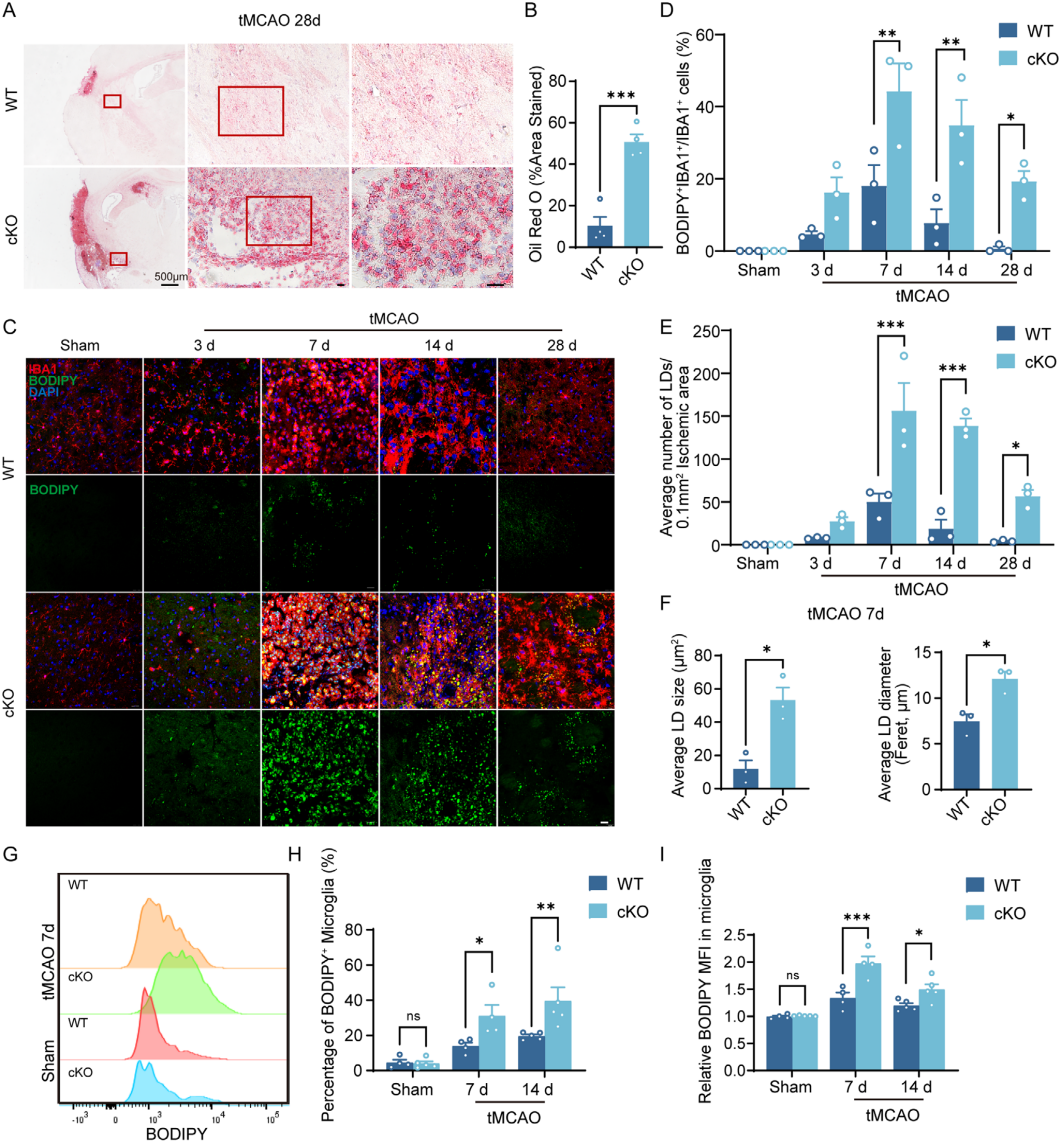
Microglial AXL alleviates lipid droplets deposition after stroke. (A,B) Representative images of oil red o staining (A) and statistics (B) of WT and AXL cKO ischemic mice at day 28 post‐tMCAO. Scale bar, 20 µm. *n* = 4 per group. (C–E) Representative immunofluorescence images of BODIPY staining (C), percentage of BODIPY^+^ microglia (D), and number of LDs (E) in the ipsilateral striatum of WT and AXL cKO mice in sham group, and at 3d, 7d, 14d, and 28d post‐tMCAO. Scale bar, 20 µm. *n* = 3 per group. (F) LD size (right) and LD diameter (left) in the ipsilateral striatum of WT and AXL cKO mice at 28d post‐tMCAO. *n* = 3 per group. (G–I) Representative flow cytometry images of MFI of BODIPY of BODIPY (G), percentage of BODIPY^+^ microglia (H), and relative BODIPY MFI (I) of WT and AXL cKO mice in sham group and at 7d post‐tMCAO. *n* ≥ 4 per group. Values were mean ± SEM. *p < 0.05, **p < 0.01, ***p < 0.001, and ns not significant.

Extensive clearance of lipid‐rich myelin debris can promote intracellular lipid droplets accumulation. Unexpectedly, abundant LDs were observed in AXL cKO mice at day 28 post‐tMCAO by Oil Red O staining (Figure 4A,B). We then profiled the dynamics of LDs in AXL cKO mice using BODIPY staining (Figure 4C–F) and flow cytometry (Figure 4G–I) across multiple time points. The LD burden in AXL cKO mice peaked at day 7 and persisted into the late phase, reflected by more LD‐positive microglia (Figure 4D), higher LD counts (Figure 4E), and larger mean LD diameter in the ischemic region (Figure 4F). Flow cytometry showed no genotype difference in LDs under sham conditions, but at 7 and 14 days post‐tMCAO, AXL cKO mice exhibited more BODIPY^+^ microglia (Figure 4H) and higher BODIPY mean fluorescence intensity (MFI) (Figure 4I) than WT. In summary, these data demonstrate that AXL deficiency leads to a profound and persistent accumulation of LDs within microglia following ischemic stroke.

### 2.5 AXL‐Deficient Microglia Display an Early Reduction in Phagocytosis of Myelin Debris, Along with Significant Later Lipid Droplet Accumulation and Lipid Peroxidation In Vitro

**FIGURE 5:**
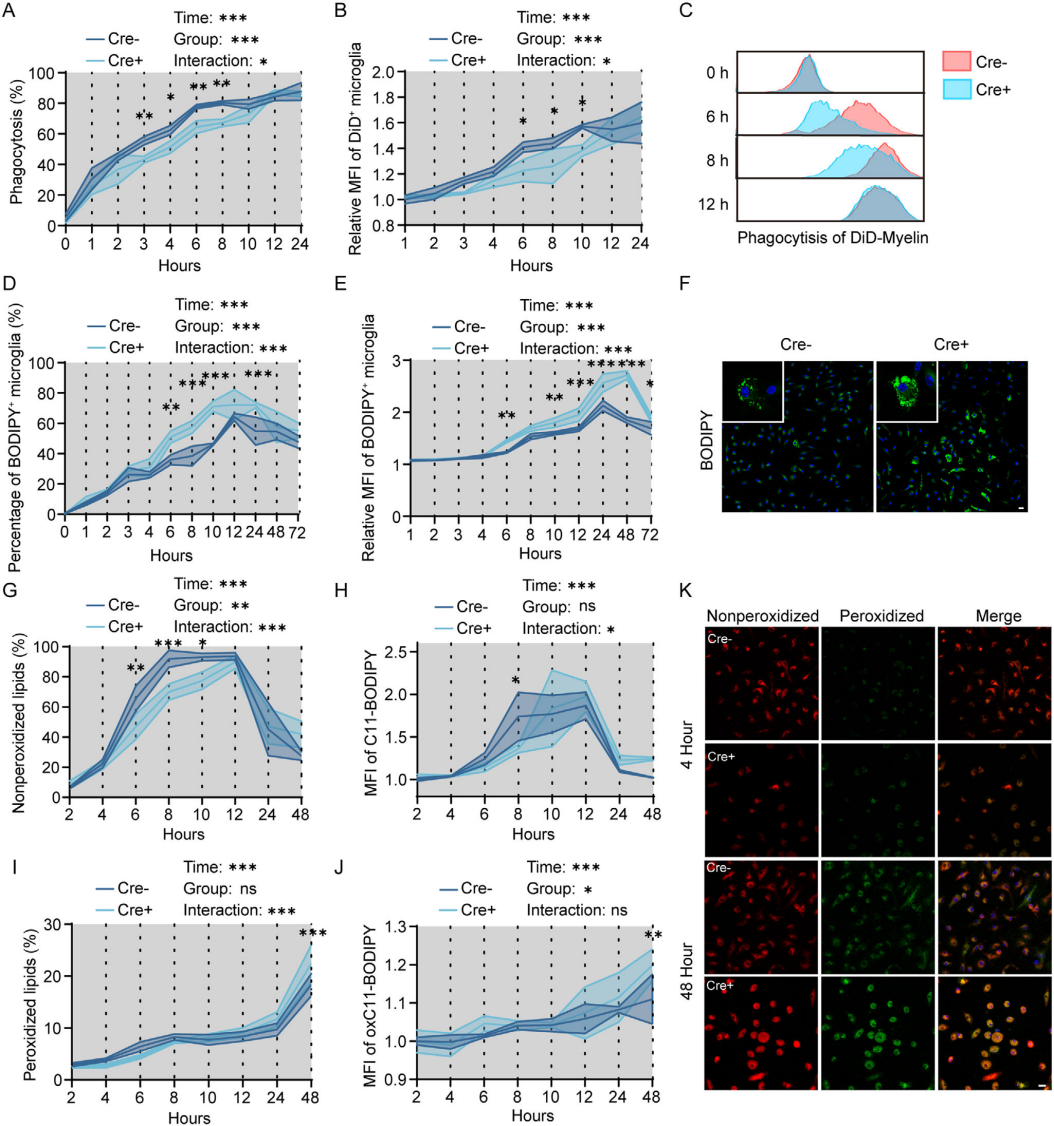
AXL‐deficient microglia display an early reduction in phagocytosis of myelin debris, along with significant later lipid droplet accumulation and lipid peroxidation in *vitro*. (A–C) The percentage (A), MFI of DID^+^ myelin debris (B) phagocytosed by Cre^+^ and Cre^−^ microglia, and representative flow cytometry images of MFI (C) after OAM. *n* = 3 per group. (D–F) The percentage (D), MFI of BODIPY (E) in Cre^+^ and Cre^−^ microglia, and representative immunofluorescence images of BODIPY (F) at 48 h after OAM. *n* = 3 per group. (G,H) The percentage of nonperoxidized lipids (G) and MFI of C11‐BODIPY (H) in Cre^+^ and Cre^−^ microglia after OAM. *n* = 4 per group. (I,J) The percentage of peroxidized lipids (I) and MFI of oxC11‐BODIPY (J) in Cre^+^ and Cre^−^ microglia after OAM. *n* = 4 per group. (K) Representative C11‐BODIPY immunofluorescence images of nonperoxidized lipids and peroxidized lipids in Cre^+^ and Cre^−^ microglia at 4 h and 48 h after OAM. *n* = 4 per group. Values were mean ± SEM. *p < 0.05, **p < 0.01, ***p < 0.001, and ns not significant.

To examine how AXL affects phagocytosis‐lipid coupling, we isolated primary microglia from AXL cKO and WT mice. Primary microglia exposed to oxygen‐glucose deprivation followed by myelin‐debris stimulation (OAM model) reproduced in vitro the inflammatory and lipid‐metabolism gene patterns seen at day 7 post‐tMCAO in vivo (Figure S2A,B). AXL loss was validated in 4‐hydroxy‐tamoxifen–treated Cre^+^ microglia versus Cre^−^ controls (Figure S2C). Early after OAM, flow cytometry revealed slower phagocytosis in Cre^+^ microglia, although both groups reached phagocytic saturation by 12 h (Figure 5A–C), suggesting compensatory phagocytic pathways activation. Given that impaired phagocytic function may trigger subsequent lipid metabolism dysregulation, we next assessed lipid droplet accumulation and lipid peroxidation levels. Notably, Cre^+^ microglia displayed increased LDs by 6 h. After replacing the medium without myelin debris at 12 h (the saturation time point), Cre^+^ microglia continued to accumulate more LDs at 24, 48, and 72 h than Cre^−^ microglia (Figure 5D–F). Then, we used C11‐BODIPY (581/591) probe to quantify lipid oxidation. By this staining probe, the peroxidized lipids showed green fluorescence while the non‐peroxidized lipids showed red fluorescence. Of particular interest was that Cre^+^ microglia exhibited a decline of non‐peroxidized lipids in the early stage (from 2 to 8 h) (Figure 5G–K), while a notable increase of peroxidized lipids in the late stage (from 24 to 48 h) after OAM (Figure 5I–K), suggesting that excessive lipid droplet accumulation leads to lipid oxidation. To determine if the observed oxidative stress was associated with a pro‐inflammatory phenotype, we analyzed cytokine levels in the culture supernatant. ELISA results confirmed that Cre^+^ cells secreted significantly higher levels of TNF‐α, IL‐6, and IL‐1β (Figure S2D). Furthermore, western blotting analysis corroborated enhanced lipid peroxidation, as evidenced by elevated 4‐hydroxynonenal (4‐HNE) expression in Cre^+^ cells (Figure S2E).

### 2.6 AXL‐Deficient Microglia Display Severe Inflammatory Response and Sphingolipid Metabolism Dysfunction by RNA‐seq

**FIGURE 6:**
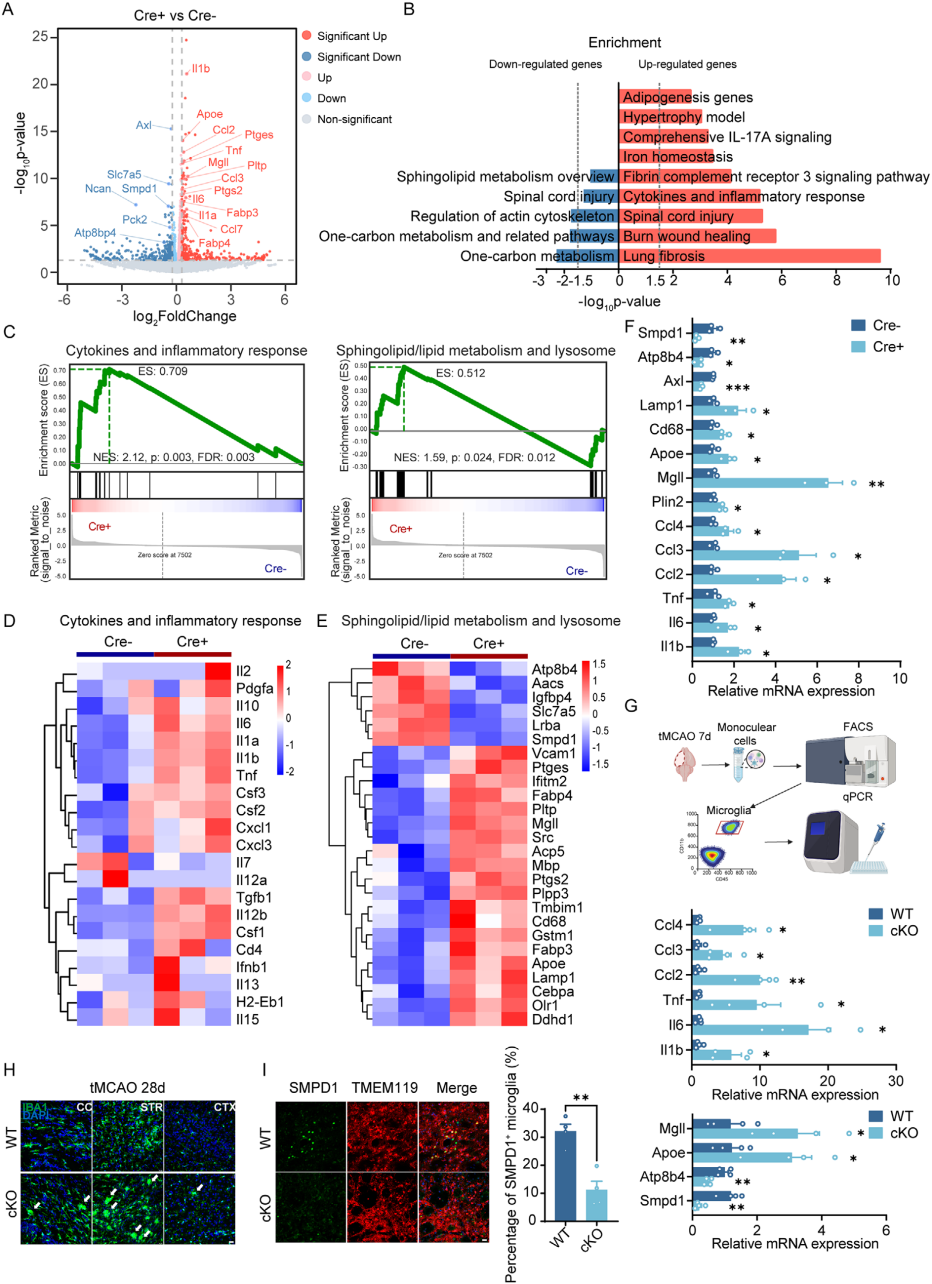
AXL‐deficient microglia display severe inflammatory response and sphingolipid metabolism dysfunction by RNA‐seq. (A) Volcano plots of all the DEGs of Cre^+^ and Cre^−^ microglia after OAM. *n* = 3 per group. (B) Top enriched pathway analysis of up‐regulated and down‐regulated genes. (C) GSEA analysis results of enrichment plot for the cytokines and inflammatory responses and sphingolipid/lipid metabolism and lysosome pathway. The NES and FDR values are shown in the plot. (D) GSEA of gene heatmap enriched in the cytokines and inflammatory response pathway. *n* = 3 per group. (E) Heatmap of sphingolipid/lipid metabolism and lysosome related genes. *n* = 3 per group. (F) QPCR detection of the expression of genes related to inflammation, chemotaxis, and lipid metabolism in Cre^+^ and Cre^−^ group. *n* = 3 per group. (G) QPCR analysis for *Il1b*, *Il6*, *Tnf*, *Ccl2*, *Ccl3*, *Ccl4*, *Smpd1*, *Atp8b4*, *Apoe*, and *Mgll* in sorted microglia from WT and AXL cKO mice at 7 days post‐tMCAO by flow cytometry. *n* = 4 per group. (H) Representative immunofluorescence images of microglia in WT and cKO mice at day 28 after tMCAO in CC (corpus callosum), STR (striatum), and CTX (cortex). White arrows showed microglial nodules. Scale bar, 20 µm. (I) Representative immunofluorescence images and statistics of SMPD1^+^ microglia in WT and AXL cKO mice at 7d post‐tMCAO. Scale bar, 20 µm. *n* = 4 per group. Values were mean ± SEM. *p < 0.05, **p < 0.01, and ***p < 0.001.

To further explore the possible mechanism of the differences between Cre^+^ and Cre^−^ microglia in myelin metabolism, we performed RNA‐seq on these two groups of microglia. Principal component analysis (PCA) revealed that the overall transcriptomic profiles were changed in Cre^+^ microglia after OAM for 6 h compared to Cre^−^ microglia (Figure S3A). There were 136 significantly differentially expressed genes (DEGs) between the two groups (Figure S3B). In Cre^+^ cells, inflammation‐related (*Il1b, Il6, Tnf*), chemotaxis‐related (*Ccl2, Ccl3, Ccl4*), lipid‐metabolism‐related (*Plin2, Mgll, Apoe, Smpd1*), and lysosome‐related (*Cd68, Lamp1*) genes were upregulated (Figure 6A). Pathway analysis indicated upregulation of “Cytokines and inflammatory response,” “Fibrin complement receptor 3 signaling pathway,” and “Comprehensive IL‐17A signaling,” with downregulation of “Sphingolipid metabolism overview,” “Regulation of actin cytoskeleton,” and “One‐carbon metabolism” in Cre^+^ microglia (Figure 6B). Gene set enrichment analysis (GSEA) showed intensified inflammatory responses and broad alterations in sphingolipid/lipid‐metabolism and lysosome‐related pathways (Figure 6C–E). Heatmaps highlighted these differences across cytokine/inflammatory, lipid‐metabolism, and lysosome genes (Figure 6D,E). qPCR validated gene‐expression changes in vitro (Figure 6F), and microglia sorted in vivo at day 7 post‐tMCAO showed consistent trends (Figure 6G). Immunofluorescence revealed more microglial nodules in AXL cKO brains at day 28 (Figure 6H)—a feature of chronic inflammation in multiple sclerosis (MS) [24] with no differences under sham conditions (Figure S3C). Notably, *Smpd1* (Sphingomyelin Phosphodiesterase 1), a key sphingolipid‐degradation gene, was markedly decreased in AXL‐deficient microglia both in vivo and in vitro (Figure 6E–G). Immunofluorescence staining also confirmed that *SMPD1* expression decreased in microglia from AXL cKO mice in vivo (Figure 6I). Together, AXL loss drives lipid‐metabolism dysfunction and chronic inflammatory activation in microglia.

### 2.7 AXL Upregulates *Smpd1* Transcription by Increasing EGR1 Expression

**FIGURE 7:**
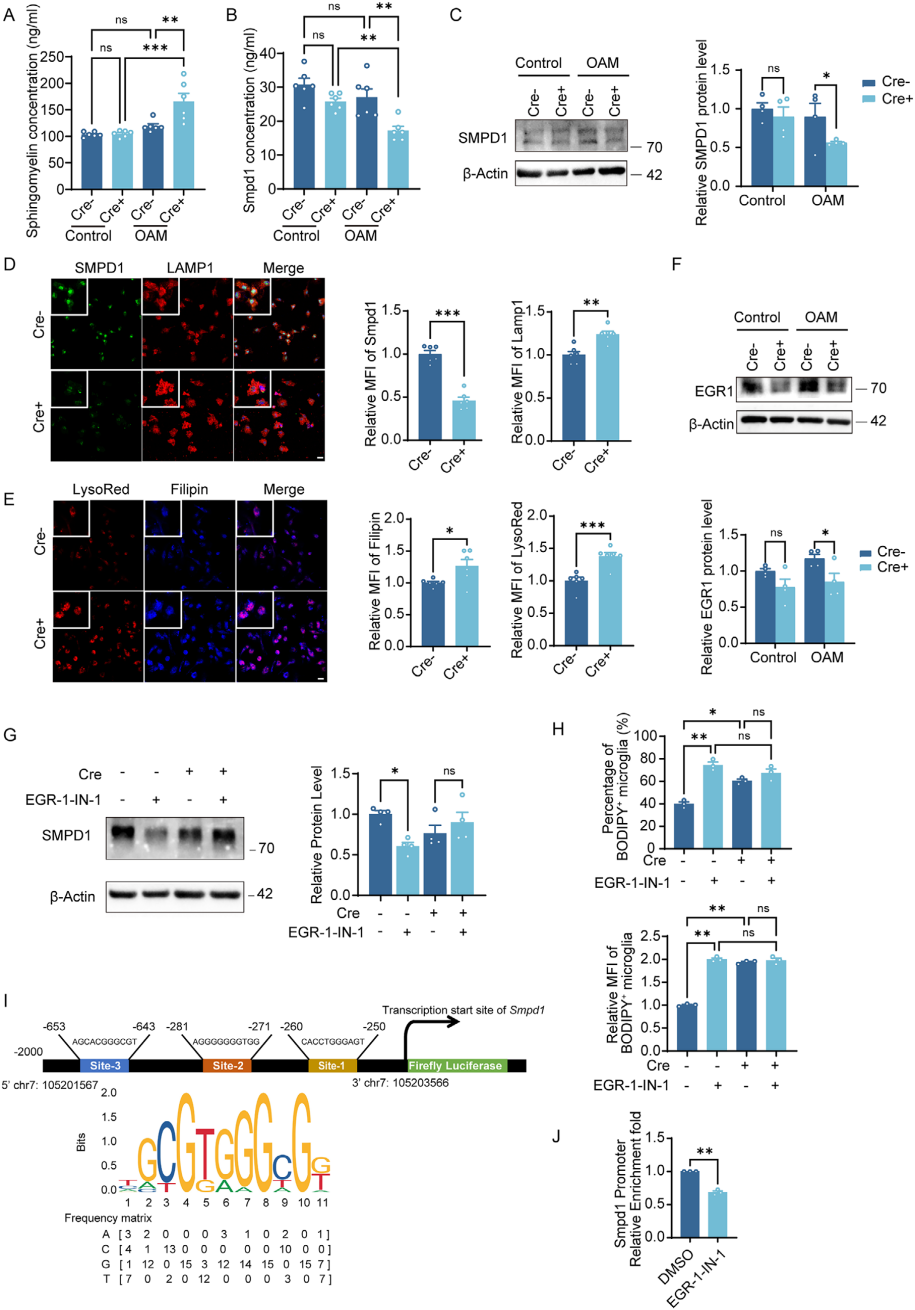
AXL upregulates *Smpd1* transcription by increasing EGR1 expression. (A, B) ELISA for sphingomyelin (A) and SMPD1 (B) in Cre^+^ and Cre^−^ microglia after OAM for 6 h. *n* = 6 per group. (C) Western blotting showed that after OAM, the SMPD1 protein level in Cre^+^ microglia decreased. *n* = 4 per group. (D) Representative immunofluorescence images (left) and MFI statistics (right) of SMPD1 and LAMP1 in Cre^+^ and Cre^−^ microglia. Scale bar, 20 µm. *n* = 6 per group. (E) Representative images (left) and MFI statistics (right) of Filipin and LysoRed staining in Cre^+^ and Cre^−^ microglia at 24 h after OAM (changing the medium at 12 h). Scale bar, 20 µm. *n* = 6 per group. (F) Western blotting showed that after OAM, the EGR1 protein level in Cre^+^ microglia decreased. *n* = 4 per group. (G) Western blotting showed that EGR‐1‐IN‐1 treatment decreased the expression of SMPD1 in Cre^−^ microglia while not in Cre^+^ microglia after OAM. *n* = 4 per group. (H) Flow cytometry analysis showed that EGR‐1‐IN‐1 treatment increased the BODIPY^+^ lipid droplets in Cre^−^ microglia after OAM for 6 h while not in Cre^+^ microglia. *n* = 3 per group. (I) Top 3 binding sites of EGR1 in the promoter sequence of *Smpd1*. (J) ChIP‐qPCR quantification of relative enrichment at *Smpd1* promoter in BV2 cells after DMSO or EGR‐1‐IN‐1 treatment after OAM for 6 h. *n* = 3 per group. Values were mean ± SEM. *p < 0.05, **p < 0.01, ***p < 0.001, and ns not significant.

*SMPD1* encodes acid sphingomyelinase (ASM), which hydrolyzes sphingomyelin, a myelin lipid, into ceramide and phosphorylcholine. Loss‐of‐function mutations in the *Smpd1* gene reduce or abolish ASM activity, impairing sphingomyelin degradation and causing lysosomal accumulation of sphingomyelin and cholesterol, a hallmark of Niemann–Pick disease [25]. To assess whether AXL influences sphingomyelin metabolism, we first confirmed reduced ASM protein levels and increased sphingomyelin accumulation in Cre^+^ microglia following OAM using ELISA (Figure 7A,B). Western blotting likewise showed decreased *SMPD1* expression in Cre^+^ microglia, particularly after OAM (Figure 7C). To further examine the role of AXL in lysosomal function, we performed lysosomal labeling using both LAMP1 immunostaining and LysoRed, a fluorescent dye that marks acidic lysosomal compartments. We observed marked lysosomal enlargement in Cre^+^ microglia and a significant decrease in *SMPD1* levels within the lysosomal compartment (Figure 7D). Notably, filipin staining revealed a substantial increase in lysosomal cholesterol deposition in Cre^+^ microglia (Figure 7E), supporting the hypothesis that AXL deficiency impairs lysosomal lipid metabolism.

To elucidate the regulatory mechanism, we tested whether AXL directly interacts with *SMPD1* by co‐immunoprecipitation but detected no binding. Transcription‐factor prediction using CHIPBase, GTRD, ChIP Atlas, and AnimalTFDB identified EGR1 as the top *Smpd1* regulator across all four databases (Figure S4A). Western blotting further showed reduced EGR1 protein in Cre^+^ microglia, suggesting that AXL influences EGR1 signaling (Figure 7F). To assess EGR1 function in this axis, we used EGR‐1‐IN‐1, a specific inhibitor of the EGR1 DNA‐binding domain, at concentrations based on a published report [26]. Treatment with EGR‐1‐IN‐1 (5 µM) significantly increased LDs formation in primary microglia in vitro (Figure S4B,C). Furthermore, EGR‐1‐IN‐1 treatment of Cre^−^ and Cre^+^ microglia demonstrated that EGR1 inhibition reduced *SMPD1* expression and increased LDs accumulation in Cre^−^ microglia after OAM, whereas Cre^+^ microglia showed no significant response (Figure 7G,H). Using the JASPAR database, we identified three putative EGR1‐binding sites within the *SMPD1* promoter, with Site‐1 and Site‐2 showing the highest prediction scores and spatial clustering (Figure 7I; Figure S4D). Chromatin immunoprecipitation followed by quantitative PCR (ChIP‐qPCR) confirmed that EGR‐1‐IN‐1 treatment significantly reduced EGR1 binding to Site‐1 and Site‐2 regions of the *SMPD1* promoter under OAM conditions (Figure 7J).

### 2.8 Increased ASM in AXL cKO Mice Alleviates Lipid Droplet Deposition and Ischemic White Matter Injury

**FIGURE 8:**
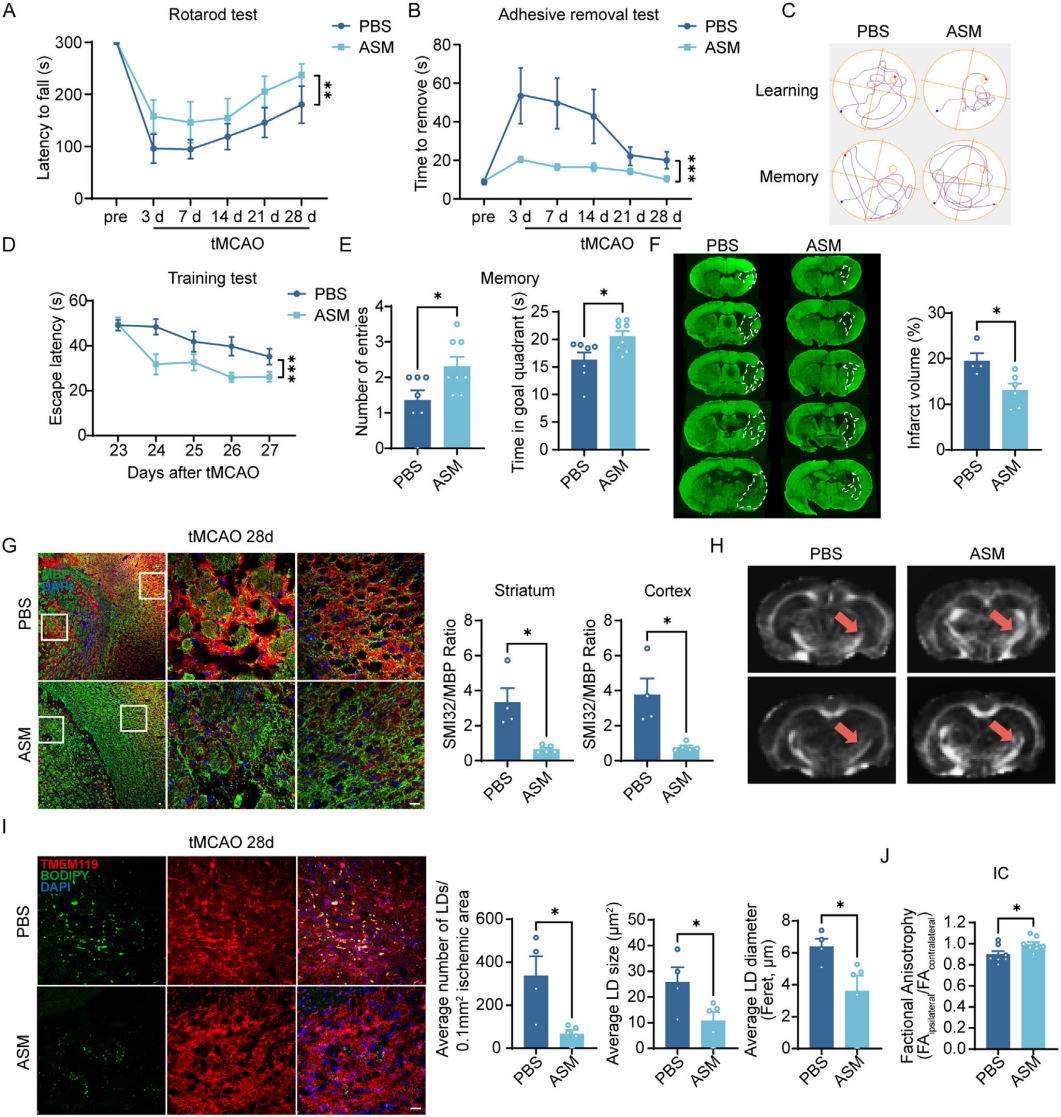
Increased ASM in AXL cKO mice alleviates lipid droplet deposition and ischemic white matter injury. (A,B) Stereotaxic injection of ASM alleviated motor and sensory coordination deficits of mice after tMCAO as assessed by rotarod test (A) and adhesive removal (B) tests. *n* = 7 for PBS group and *n* = 9 for ASM group. (C–E) Representative images of swim paths of PBS and ASM treatment mice (C), training test for learning (D), and probe test (E) for memory to evaluate cognitive functions through Morris water maze. *n* = 7 for PBS group and *n* = 8 for ASM group. (F) Representative images and statistics of MAP2 staining. *n* ≥ 4 per group. (G) Immunofluorescence staining and statistics of SMI32 and MBP in the cortex and the striatum area of mice with PBS and ASM treatment. *n* ≥ 4 per group. (H) Representative FA maps of mice with PBS and ASM treatment. Red arrowheads pointed IC. (I) Representative immunofluorescence images of BODIPY staining, number of LDs, LD size, and LD diameter in the ipsilateral striatum of AXL cKO mice after PBS and ASM treatment at 28d post‐tMCAO. Scale bar, 20 µm. *n* ≥ 4 per group. (J) Quantification of FA value expressed as the ratio of ipsilateral values to the contralateral values in the IC area. *n* = 7 for PBS group and *n* = 8 for ASM group. Values were mean ± SEM. *p < 0.05, **p < 0.01, ***p < 0.001, and ns not significant.

**FIGURE 9:**
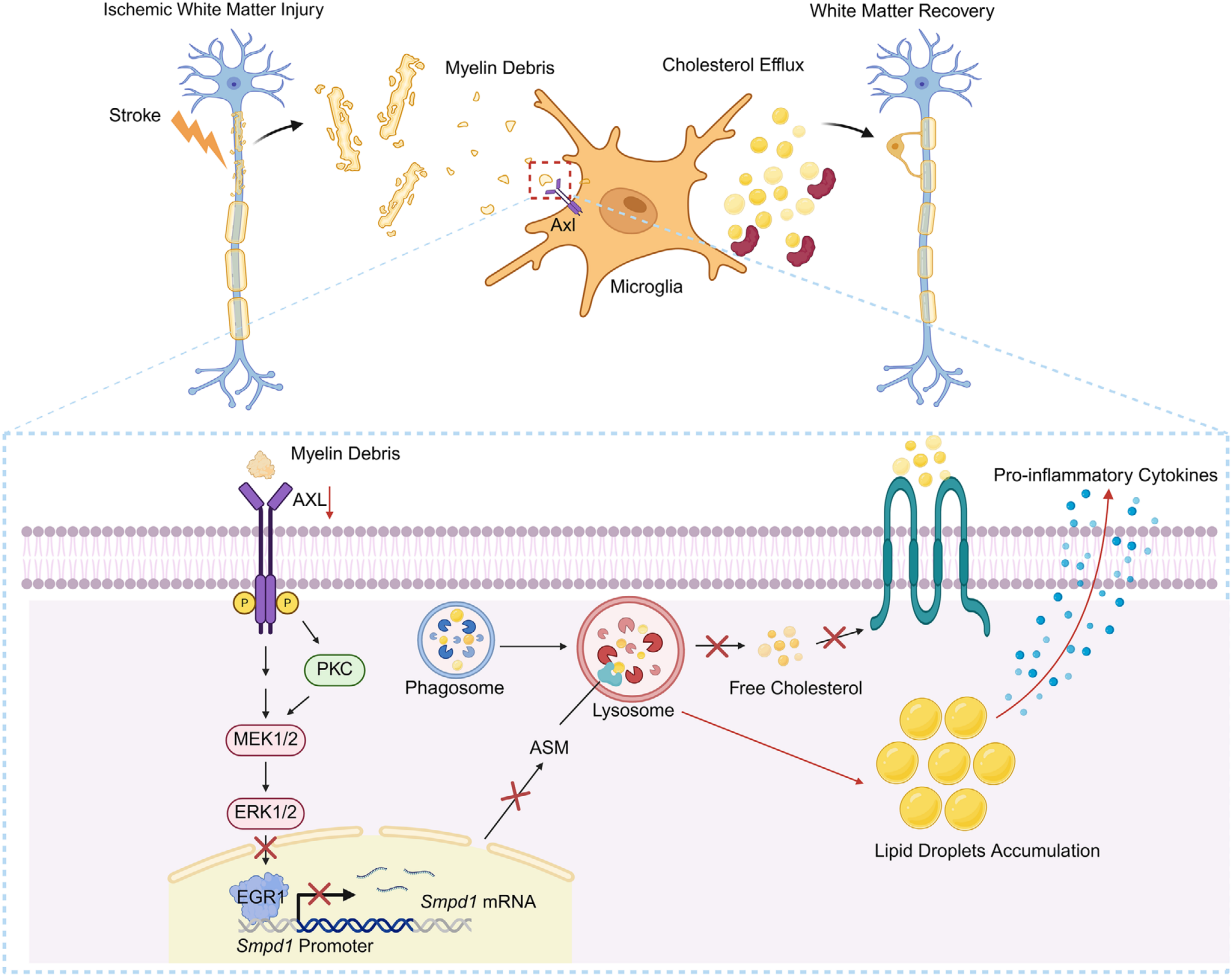
Schematic of AXL‐mediated white matter repair via suppression of microglial lipid droplet accumulation post‐ischemic stroke. Following ischemic stroke, myelin debris generated from injured white matter is phagocytosed by microglia via the AXL receptor. Upon engaging myelin‐derived lipids, AXL activates the downstream PKC or MEK/ERK signaling axis, which in turn phosphorylates and activates the transcription factor EGR1. EGR1 then binds to the *Smpd1* gene promoter, leading to increased expression of ASM. ASM hydrolyzes sphingomyelin to ceramide, which prevents excessive lipid droplet accumulation and the associated pro‐inflammatory response, thereby promoting myelin debris clearance and creating a regenerative environment that supports white matter recovery.

We next investigated whether ASM supplementation in AXL cKO mice could rescue LDs deposition and myelin injury after stroke. Guided by prior studies, we stereotaxically injected recombinant mouse ASM into AXL cKO mice immediately after tMCAO [27, 28]. The experimental timeline is shown in Figure S5A. Immunofluorescence stanning and western blot analyses demonstrated that a single stereotaxic injection of ASM significantly increased ASM levels in the right cerebral hemisphere for at least 7 days (Figure S5B,C). Mortality after tMCAO did not differ between mice receiving sterile PBS or ASM injected into the right lateral ventricle (Figure S5D). In behavioral tests, rotarod and adhesive removal assays showed that ASM‐treated mice stayed on the rotarod longer and removed the adhesive faster, indicating better recovery of motor and sensory coordination after tMCAO (Figure 8A,B). In the MWM at 4 weeks post‐tMCAO, ASM‐treated mice exhibited improved spatial learning and memory relative to PBS controls, as reflected by a shorter time to find the platform during training (Figure 8C,D), more platform crossings, and longer time in the target quadrant during testing (Figure 8E). Swimming speed did not differ between groups (Figure S5E). MAP2 staining showed that infarction and atrophy volumes were significantly smaller in the ASM group 28 days after tMCAO (Figure 8F). The SMI32/MBP ratio was lower in the cortex and striatum in ASM‐treated mice than in PBS controls (Figure 8G). MRI indicated an increased FA ratio and decreased MD ratio in the ASM group compared with PBS in the IC region (Figure 8H,J; Figure S5F), consistent with attenuated ischemic white‐matter injury in the striatum. LDs deposition in the striatum was also reduced in ASM‐treated mice (Figure 8I), and *SMPD1* expression increased in microglia (Figure S5G). We additionally evaluated the therapeutic time window and found that administering ASM 24 h after tMCAO improved acute‐phase behavioral outcomes—better modified Neurological Severity Score (mNSS), rotarod, and adhesive removal performances—though survival was unchanged (Figure S5H–K). Delayed administration also significantly reduced LDs accumulation at 7 days post‐tMCAO in AXL cKO mice, decreasing LD number, volume, and diameter (Figure S5L), which suggested that regulating LDs formation exceeding the hyperacute phase (e.g., >24 h) after stroke may be a feasible therapeutic strategy. In summary, intraventricular ASM supplementation alleviated post‐stroke white‐matter injury and LDs deposition caused by microglial AXL deficiency. A schematic overview is shown in Figure 9.

## 3 Discussion

To summarize, AXL expression is markedly upregulated in microglia after ischemic stroke, helping mitigate ischemic white‐matter damage and improve prognosis. AXL‐deficient microglia show reduced phagocytic capacity and increased lipid droplets accumulation, leading to myelin debris deposition, lipid peroxidation, and a heightened inflammatory response. Mechanistically, AXL regulates *Smpd1* transcription by modulating EGR1, a key pathway underlying these pathological changes. Collectively, our results identify microglial AXL as a central regulator of myelin repair after ischemic stroke.

AXL belongs to the TAM receptor family and has diverse roles in phagocytosis, tissue repair, inflammation, cell proliferation, and migration [29]. Under physiological conditions, AXL expression is minimal in resting microglia but becomes markedly upregulated in response to inflammation and injury, including intracerebral hemorrhage, AD, and MS [30]. Interest in AXL's role in demyelinating diseases is growing. In aged white matter, AXL is abundantly expressed on microglia, which often aggregate into nodules and actively clear degenerated myelin debris [10]. In MS, AXL promotes remyelination by clearing myelin debris and dampening neuroinflammation, highlighting a potential therapeutic target [30, 31]. After subarachnoid hemorrhage, activation of AXL signaling reduces white‐matter injury [32]. Building on this evidence, we show that AXL expression is robustly and persistently upregulated in microglia following ischemic stroke and that this upregulation mitigates white‐matter damage and improves outcomes through 28 days after tMCAO. Beyond myelin protection, AXL also has acute neuroprotective effects: at 24 h after tMCAO in rats, Gas6/AXL activation attenuates ischemic injury by suppressing the TLR/TRAF/NF‐κB cascade [13]. Clinically, lower serum AXL levels are associated with a higher risk of hemorrhagic transformation after intravenous thrombolysis in stroke patients [33]. Together, these findings support a crucial role for AXL in post‐stroke myelin repair.

In neurodegenerative and demyelinating diseases, AXL drives microglial phagocytosis and clearance of substances such as Aβ, apoptotic neurons, and myelin debris, which is crucial for maintaining white matter injury and promoting remyelination [29]. Consistently, we observed impaired phagocytic activity and excessive myelin‐debris accumulation in AXL cKO mice after tMCAO. Sensing and clearing myelin‐debris signals is therefore an initial step by which AXL improves the microenvironment for white‐matter repair. Increasing evidence indicates that phagocytosis of myelin debris can disrupt lipid homeostasis in phagocytes [14, 34, 35]. Notably, phagocytic receptors regulate not only engulfment but also intracellular lipid metabolism [18, 36, 37]. For example, TREM2 deletion in mice impairs microglial phagocytosis while increasing intracellular lipid accumulation, indicating that defective phagocytosis may exacerbate, rather than relieve, lipid burden [18]. After stroke, TREM2‐deficient microglia show reduced phagocytosis and cholesterol‐ester accumulation, likely driven by enhanced lipid synthesis with diminished cholesterol efflux and lipid hydrolysis, ultimately promoting a pro‐inflammatory phenotype [19]. Pharmacologic TREM2 activation by fingolimod facilitates cholesterol transfer from microglia to oligodendrocytes, promoting remyelination and ameliorating chronic ischemic white‐matter injury [38]. Analogously, we found that AXL‐deficient microglia develop pronounced lipid droplets accumulation reminiscent of macrophage foam cells in atherosclerosis, suggesting that microglial AXL accelerates not only myelin‐debris clearance but also its intracellular processing and lipid metabolism.

Lipid droplets are neutral‐lipid organelles (triglycerides and cholesterol esters) that increase with aging, inflammation, and stress [39, 40]. In AD models, lipid‐droplet‐rich microglia are phagocytosis‐defective, generate high reactive oxygen species, and secrete pro‐inflammatory cytokines [41]; these lipid‐laden microglia also express less AXL [17]. Similarly, microglial lipid‐droplet accumulation increases 3–7 days after MCAO, and these cells display a robust pro‐inflammatory phenotype [42]. We likewise observed elevated inflammatory‐factor secretion in AXL‐deficient microglia. Prominent microglial nodules were present in the corpus callosum and striatum of the ischemic hemisphere (hallmarks of demyelinating pathology), where they are enriched in white matter and predominantly pro‐inflammatory [43]. Together, these findings support a model in which phagocytic receptors such as AXL regulate not only engulfment but also downstream digestion, lipid recycling, and resolution of inflammation. Although our study focused on acute focal ischemia mediated white matter injury, the AXL‐mediated reparative mechanism identified here may hold potential significance for multiple white matter injury diseases, especially in cerebral small vessel diseases (CSVD). In CSVD, white matter damage is one of the key pathological mechanisms. On a molecular level, the abnormal phagocytic or lysosomal abilities of microglia may exacerbate white matter damage in CSVD [4, 10, 44]. Therefore, the Axl‐mediated white matter repair by microglia may have a certain degree of universality and more clinical application scenarios, which will be further verified in our subsequent work.

Currently, the molecular mechanisms linking AXL to biology of lipid droplet remain largely unknown. Existing studies indicate that phagocytic receptors regulate cellular lipid metabolism through multiple mechanisms, such as activating the transcription factor LXR to promote cholesterol efflux or modulating lysosomal‐membrane permeabilization and lysosomal function to influence lipid degradation and redistribution [45, 46, 47]. Our transcriptomic analysis suggested that sphingolipid metabolism is perturbed in AXL‐deficient microglia. Among the altered genes, *Smpd1* expression was markedly decreased, and sphingomyelin levels were elevated in AXL‐deficient microglia. *Smpd1* encodes ASM, a key lysosomal enzyme that converts sphingomyelin into ceramide and phosphorylcholine. ASM deficiency, as in Niemann–Pick disease types A and B, leads to sphingomyelin and cholesterol accumulation, lysosomal dysfunction, and secondary LDs formation [48, 49]. Sphingomyelin is a critical regulator of endolysosomal lipid digestion; its accumulation can inhibit cholesterol transfer and impair lysosomal function [50]. Using LAMP1 immunofluorescence and LysoRed to trace lysosomes, AXL‐deficient microglia exhibited increased lysosome number and enlargement, along with elevated cholesterol deposition, indicating lysosomal impairment reminiscent of Niemann–Pick disease. Lysosomes degrade LDs through lipophagy, an essential mechanism for maintaining intracellular balance of LDs [51]. Prior work shows that ASM deficiency impairs degradation of myelin‐derived lipids, causing excessive LDs accumulation and microglial dysfunction [22], whereas recombinant ASM restores lipid clearance and reduces LDs accumulation in deficient models [28, 52]. These observations support a model in which AXL deficiency compromises lysosomal function, reducing lipophagy and thereby increasing LDs deposition via reduced ASM activity. Consistent with this, administration of recombinant ASM to AXL cKO mice after stroke alleviated functional deficits and attenuated white‐matter damage, supporting a compensatory role for ASM in lysosomal lipid processing when AXL is absent. The efficacy of ASM given 24 h after tMCAO can be explained mechanistically by coverage of peak lipid dysregulation spanning the acute and subacute phases, intervening at a critical point in pathological evolution. Although the roles of ASM and its downstream product ceramide in stroke are complex and context‐dependent [53, 54, 55], our findings reinforce the concept that targeting post‐stroke lipid metabolism is pivotal for functional recovery. Thus, ASM administration may act not merely as replacement therapy but as a strategy to recalibrate dysregulated sphingolipid pathways during the critical repair phase after ischemic injury. To further delineate how AXL regulates *Smpd1* expression, we found that AXL up‐regulates *Smpd1* transcription by activating EGR1. EGR1 has been implicated as a transcription factor for *Smpd1* in human leukemia and colon cancer cell lines [56], and Gas6‐dependent AXL activation upregulates the early gene Egr1 in murine pituitary gonadotrope [57]. In our microglial system, an EGR1 inhibitor reduced EGR1 binding to the *Smpd1* promoter by ChIP‐qPCR, confirming EGR1‐mediated transcriptional control. Regarding *Smpd1* levels and LDs deposition, the EGR1 inhibitor produced no additional effect in AXL‐deficient microglia, suggesting that AXL regulates *Smpd1* via the EGR1 pathway. Upon activation, the AXL tyrosine‐kinase domain undergoes conformational change that facilitates receptor dimerization and autophosphorylation, initially at Y698, Y702, and Y204, and subsequently at Y779, Y821, and Y866, recruiting downstream effectors and activating pathways including MEK/ERK and protein kinase C (PKC), which can induce EGR1 [58, 59]. While our current study focused on the upregulation of EGR1 protein levels and its transcriptional regulation of *Smpd1*, previous studies have established that the PKC/MEK/ERK pathway promotes EGR1 phosphorylation and nuclear translocation, which is essential for its transcriptional activity [60, 61]. Furthermore, given that EGR1 is a widely expressed immediate‐early gene, it is important to note that the specificity of this regulation within microglia is likely conferred by the upstream receptor AXL. Since AXL is preferentially expressed or activated in microglia under these pathological conditions [62], it channels the ubiquitous EGR1 signaling specifically toward *Smpd1*‐mediated lipid metabolism in this cell type, thereby minimizing potential off‐target effects associated with EGR1's broad expression in other tissues. Beyond EGR1, *Smpd1* transcription can be influenced by additional factors: SP1 contributes to basal *Smpd1* expression in macrophages [63] (also identified in our TF predictions), and the lysosomal master regulator TFEB directly regulates *Smpd1* expression and promotes lysosomal exocytosis [64]. However, our observation that pharmacological inhibition of EGR1 alone is sufficient to induce LDs accumulation mimics the phenotype of AXL deficiency. This suggests that, in the specific context of our study, the EGR1 pathway serves as the key and primary mechanism governing *Smpd1*‐mediated lipid metabolism, playing a more dominant role than other basal regulators.

In conclusion, our findings highlight the protective role of microglial AXL in ischemic white‐matter injury. AXL facilitates myelin‐debris clearance and regulates LD metabolism, thereby modulating chronic inflammation and enhancing functional recovery after stroke. These insights reveal mechanistic and therapeutic avenues for post‐stroke neurorehabilitation.

## 4 Experimental Section

### 4.1 Animals

Healthy male C57BL/6J mice (6–8 weeks, 20–25g), microglia‐specific AXL knockout mice (*Axl^flox/flox^*; *Cx3cr1^CreERT2^*), and wild‐type littermates (*Axl^flox/flox^*) were obtained from the Model Animal Research Center of Nanjing University (China). The animals were housed in specific pathogen‐free conditions, with a controlled temperature maintained at 22°C–25°C and humidity at 50%–60%. A standardized 12‐h light/dark cycle was implemented, and the mice were provided ad libitum access to food and water throughout the experimental period.

### 4.2 Murine Model of Ischemic Stroke

Healthy male mice (8–10 weeks old, 20–25 g) were subjected to either transient middle cerebral artery occlusion (tMCAO) or sham surgery was performed in as previously described [23]. The tMCAO model effectively simulates unilateral focal cerebral ischemia, with its favorable survival rate permitting extended ischemic durations suitable for our investigation. To induce microglia‐specific AXL knockout mice (*Axl^flox/flox^*; *Cx3cr1^CreERT2^*) and wild‐type littermate controls (*Axl^flox/flox^*) received intraperitoneal injection of tamoxifen citrate (MCE, HY‐13757, 20 mg/mL) for five consecutive days. Experimental procedures commenced three weeks post‐tamoxifen treatment. Mice were anesthetized with Avertin (Sigma‐Aldrich, 100–200 µL/10 g, i.p.) and maintained at 37.5°C ± 0.5°C throughout surgery using a heating pad. Following neck incision, the middle cerebral artery (MCA), external carotid artery, and internal carotid artery (ICA) were isolated. A 6‐0 nylon suture (Doccol Corporation, MA, USA) was introduced into the ICA via the common carotid artery bifurcation and advanced into the MCA. Cerebral blood flow was monitored using laser Doppler flowmetry (Perimed Corporation) through a temporal incision until ipsilateral MCA perfusion decreased to ≤ 30% baseline. After 60 min of occlusion, the filament was removed to permit reperfusion. Sham‐operated mice underwent identical procedures without filament insertion.

### 4.3 Stereotaxic Intracranial Injection of Recombinant Mouse *SMPD1* (r*SMPD1*)

Stereotaxic injections were performed as previously described [23]. Briefly, male microglia‐specific AXL knockout mice were immobilized in a stereotactic frame immediately after transient middle cerebral artery occlusion (tMCAO). Ophthalmic ointment was applied to prevent corneal dehydration. A midline scalp incision exposed the skull, and the injection site was localized using stereotactic coordinates (1.0 mm lateral to the right side of bregma, 0.22 mm posterior to bregma, 3.0 mm ventral to bregma). Recombinant mouse *SMPD1* protein (MCE, #HY‐P76134, 1 µg in 2 µL) or an equal volume of sterile PBS was injected into the right lateral ventricle at a rate of 50 ng/min. To minimize reflux, the microelectrode was retained for 10 min post‐injection. The incision was sutured, and mice recovered on a thermostatic heating pad. Behavioral tests were conducted before and 4 weeks post‐injection. Mice without pre‐surgical behavioral differences were included in further analyses.

### 4.4 Neurological Behavioral Assessments

Rotarod Test: Motor coordination was evaluated using a five‐lane rotarod (IITC Life Science) at 20, 30, and 40 rpm for 5 min each training. Mice were trained twice daily for 3 days (15‐min inter‐trial interval) pre‐surgery. On test day, latency to fall was averaged across three trials.

Adhesive Removal Test: Sensory‐motor deficits were assessed by applying 2 × 3 mm adhesive strips to forepaws. Mice were trained once daily for 3 days pre‐surgery. Mice were trained once daily for 3 days. On test sessions, the time to remove strips were recorded within a 2‐min cutoff.

Morris Water Maze (MWM): Spatial memory was assessed on post‐tMCAO day 28 [65]. During acquisition (Days 1–5), mice underwent four trials per day. In each daily session, every mouse was released into the pool facing the wall from all four quadrants, with an inter‐trial interval of at least 15 min. Mice had 60 s to locate a submerged platform. Upon successfully locating the platform, mice were conditioned to remain on it for 30 s. If a mouse failed to locate the platform within the allotted time, it was allowed to rest on the platform for 30 s, with latency time recorded as 60 s. On Day 6 (probe trial), the platform was removed, and mice swam for 60 s from two diagonal start points. Data were analyzed using ANY‐maze software (Stoelting, USA).

### 4.5 Magnetic Resonance Imaging (MRI)

A 9.4T Bruker MR system (BioSpec 94/20 USR, Bruker) with an 86 mm volume coil and surface coil was used. Anesthetized mice (2.5%–3% isoflurane) were stabilized on a platform with ear/dental bars.

T2‐weighted imaging: Rapid acquisition with relaxation enhancement (RARE) sequence: repetition time (TR) = 2,500 ms, echo time (TE) = 33 ms, field of view (FOV) = 20 × 20 mm^2^, matrix = 256 × 256, 22 slices (0.7 mm thickness).

Diffusion tensor imaging (DTI): Spin‐echo EPI with b‐values = 0/1,000 s/mm^2^ (30 non‐collinear directions); parameters: δ = 4.1 ms, Δ = 10.3 ms, TR = 1,500 ms, TE = 23.27 ms, FOV = 20 × 20 mm^2^, matrix = 128 × 128, 22 slices (0.7 mm thickness).

Data were converted to NIFTI (MRIcron) and processed via FSL v5.0.9 for eddy/motion correction and fractional anisotropy (FA) map generation.

### 4.6 Electron Microscopy (EM)

Mice were perfused transcardially with PBS post‐anesthesia. Target tissues were dissected into 1 mm^3^ blocks, fixed in EM‐grade glutaraldehyde (overnight), and post‐fixed with 2% osmium tetroxide (2 h). After ethanol dehydration, samples were embedded in epoxy resin. Ultrathin sections (70 nm) were cut (Leica UC7 ultramicrotome), stained with 2% uranyl acetate (8 min, dark) and 2.6% lead citrate (8 min, CO_2_‐free), and imaged on a Hitachi HT7800 TEM.

### 4.7 Fluorescence‐Activated Cell Sorting (FACS) and Flow Cytometry

Mice were euthanized via cervical dislocation at different timepoints after tMCAO. The right cerebral hemisphere was dissected, excluding the brainstem and olfactory bulb to focus on cortical regions. Tissue was minced with sterile scissors and homogenized in PMG buffer using a mechanical grinder or glass homogenizer. The homogenate was filtered through a 70 µm cell strainer to obtain a single‐cell suspension. Cells were subjected to stratification using a 30%–70% Percoll gradient (GE Healthcare BioSciences, NJ, USA), followed by centrifugation with controlled slow acceleration and deceleration at 2, 500 rpm for 20 min. The interphase fraction was collected, resuspended in PMG, and centrifuged. Cells were harvested and incubated in the dark at 4°C for 30 min with a combination of antibodies: anti‐CD45 (BioLegend, #103114, 1:1000), anti‐CD11b (Invitrogen, #2513551, 1:500), CD68 (Biolegend, #333809, 1:300).and BODIPY 493/503 (Cayman, #25892‐10, 1:10000). After terminating staining with PBS, cells were resuspended by centrifugation. The cells were resuspended and CD11b^+^CD45^int^ microglia were isolated using a BD FACS system (BD Biosciences, Carlsbad, CA, USA) for downstream analyses.

### 4.8 Immunofluorescence Staining and 3D Reconstruction

Mice were anesthetized and transcardially perfused with phosphate‐buffered saline (PBS) followed by 4% paraformaldehyde (PFA). Brains were post‐fixed overnight at 4°C, then cryoprotected in 15% and 30% sucrose (24 h each). Coronal sections (20 µm) were cut using a cryostat (Leica, Wetzlar, Germany). Sections were permeabilized (0.25% Triton X‐100, 20 min, RT), blocked with 2% bovine serum albumin (BSA, 1.5–2 h), and incubated with primary antibodies overnight at 4°C. After washing three times with PBS, sections were incubated with secondary antibodies (2h, RT, dark), then the cell nuclei were stained with 4’, 6‐diamidino‐2‐phenylindole (DAPI, 5 mg/mL, Bioworld Biotechnology) for 15 min. Cultured cells were fixed in 4% PFA (20 min, RT) before identical staining procedures. Images were acquired using a confocal fluorescence microscope (Olympus FV3000, Japan). All images were captured under the same microscope configuration and processed using the same adjustments and parameters to ensure consistency across the dataset. The exported images were imported into ImageJ (NIH) for analysis, where quantification was performed by two independent observers who were blinded to the experimental groups. Three‐dimensional reconstructions of the images were generated using the Imaris image processing software.

Primary antibodies used included anti‐APC (Calbiochem, #OP80, 1:500), anti‐AXL (R&D systems, #AF854, 1:300), anti‐Iba1 (Abcam, #ab5076, 1:500), anti‐TMEM119 (Synaptic Systems, #400011, 1:500), anti‐dMBP (Millipore, #ab5864, 1:200), FluoroMyelin (Invitrogen, #F34651, 1:1000), anti‐MBP (Abcam, #ab7349, 1:500), anti‐Map2 (Bioworld, #BS3487, 1:100), anti‐Pdgfα (CST, #3174s, 1:500), anti‐SMI32 (BioLegend, #801702, 1:500), anti‐*Smpd1* (Proteintech, #14609‐1‐AP, 1:300), anti‐Human CD107a/Lamp1 (Proteintech, #65051‐1‐Ig, 1:300), anti‐Lamp1 (Abcam, #ab24170, 1:500).

### 4.9 Oil Red O Staining

Oil Red O staining was performed using a modified staining kit (Beyotime, #C0158S) following the manufacture's protocol. Briefly, brain sections were incubated with staining wash solution for 20 s to cover the samples. The wash solution was then removed, and sections were treated with freshly prepared Oil Red O working solution for 10‐20 min. After incubation, the working solution was discarded, and sections were rinsed with staining wash solution for 30 s. Subsequently, sections were immersed in distilled water and agitated on a shaker for 20 s to ensure thorough washing. Images were acquired using an upright microscope (Olympus IX73, Japan).

### 4.10 Preparation and Fluorescent Labeling of Myelin Debris

Myelin debris was prepared as previously described [65, 66]. Briefly, after euthanasia, mouse brains were dissected and placed in 0.32 m sucrose solution. The tissue was minced into approximately 5 mm^3^ fragments, transferred to a 50 mL conical tube, and homogenized using a sterile rotor homogenizer. The homogenate was diluted with 0.32 m sucrose solution and layered onto 0.83 M sucrose in a thin‐walled ultracentrifuge tube. After ultracentrifugation (100 000 g, 45 min, 4°C, minimal acceleration/deceleration), crude myelin debris was collected from the interface, resuspended in Tris·Cl buffer, and homogenized again. The suspension was centrifuged (100 000 g, 45 min, 4°C, maximal acceleration/deceleration), and the pellet was resuspended in Tris·Cl buffer. Following a second centrifugation, the pellet was resuspended in sterile PBS to the final concentration of 100 mg/mL), aliquoted into pre‐weighed tubes, and stored at −80°C. For fluorescent labeling, myelin debris (1 mg/mL) was stained with DiD or DiO (Beyotime, China) and added into microglia for subsequent immunofluorescence or flow cytometry.

### 4.11 Primary Microglia Culture and Treatment

The cerebral cortex was from 1‐2‐day‐old C57BL/6J pups, microglia‐specific AXL knockout pups, and wild‐type pups. After digestion and resuspension in culture medium, primary microglia were isolated. The isolated primary microglia were cultured in PMG, which is composed of 90% Dulbecco's Modified Eagle Medium (DMEM, Invitrogen, Frederick, MD, USA), 10% fetal bovine serum (FBS, Hyclone, Logan, UT, USA), and 100 U/mL antibiotics, in a humid atmosphere of 37°C and 5% CO_2_ for 10–13 days. Following this incubation period, the primary microglia were carefully collected by gentle shaking and subsequently plated into 12‐well or 24‐well plates for subsequent experimental analyses. 4‐hydroxy‐tamoxifen (MCE, HY‐16950) was added to culture media at 2 µM for 48 h to induce deletion of AXL in cultured microglia from microglia‐specific AXL knockout pups and wild‐type pups.

### 4.12 Oxygen–Glucose Deprivation (OGD) and Reperfusion with Myelin Debris Stimulation

Primary microglia were seeded in 12‐ or 24‐well plates. After attachment, cells were subjected to OGD by replacing the culture medium with glucose‐free DMEM (Invitrogen, USA) and incubating in a hypoxic chamber (5% CO_2_, 95% N_2_, 37°C) for 4 h. Following OGD, cells were replenished with normal growth medium (DMEM with 10% FBS and 100 U/mL antibiotics) and stimulated with myelin debris (1 mg/mL). Cells were harvested at designated timepoints for downstream analyses.

### 4.13 In Vitro Myelin Debris Uptake Assay

Myelin debris uptake was assessed using DiD‐ or DiO‐labeled debris (Beyotime, China). After 4 h OGD, microglia were treated with DiD‐myelin debris (1 mg/mL) and analyzed at reperfusion timepoints via flow cytometry (BD Accuri C6).

### 4.14 Lipid Droplet, C11‐BODIPY 581/591, Lysotracker, and Filipin Staining

BODIPY staining: For immunofluorescence, cells were incubated with BODIPY (1:1000 in PBS, 30 min) after secondary antibody treatment. For flow cytometry, BODIPY (1:10000) was co‐stained with other markers (4°C, 30 min).

C11‐BODIPY 581/591: Cells were washed with warm PMG, stained with C11‐BODIPY (5 µM, 37°C, 30 min), and analyzed via flow cytometry (FITC/PE channels) or confocal microscopy (Olympus FV3000, Japan).

LysoTracker Red: Cells were stained with LysoTracker Red (50 nM, 37°C, 2 h), washed, and protected from light.

### 4.15 Filipin Staining

Fixed brain sections or cells were stained with filipin (0.05 mg/mL, 2 h, dark) and washed 3 times with PBS.

### 4.16 RNA Extraction and Quantitative Real‐Time PCR

Total RNA was isolated using TRIzol (AG, China) and reverse‐transcribed into cDNA (Vazyme, China). qPCR was performed with SYBR Green (Vazyme, China), using β‐actin as the reference gene. Primer sequences are listed below.

*Axl*   Forward    GGAACCCAGGGAATATCACAGG
     Reverse    AGTTCTAGGATCTGTCCATCTCG
*Apoe*  Forward   CTGACAGGATGCCTAGCCG
     Reverse    CCAGCTCCTTTTTGTAAGCCTTT
*Atp8b4*  Forward   GAGAAGTTCCAGTATGCGGAC
     Reverse   TGACAGCCGTCATCGAGATCA
*Il1b*  Forward   AAGCCTCGTGCTGTCGGACC
     Reverse   TGAGGCCCAAGGCCACAGG
*Il6*   Forward   GCTGGTGACAACCACGGCCT
     Reverse   AGCCTCCGACTTGTGAAGTGGT
*Tnf*   Forward   CAAGGGACAAGGCTGCCCCG
     Reverse   GCAGGGGCTCTTGACGGCAG
*Ccl2*  Forward   TTAAAAACCTGGATCGGAACCAA
     Reverse   GCATTAGCTTCAGATTTACGGGT
*Ccl3*  Forward   TTCTCTGTACCATGACACTCTGC
     Reverse    CGTGGAATCTTCCGGCTGTAG
*Ccl4*  Forward    TTCCTGCTGTTTCTCTTACACCT
     Reverse    CTGTCTGCCTCTTTTGGTCAG
*Mgll*   Forward   CGGACTTCCAAGTTTTTGTCAGA
     Reverse   GCAGCCACTAGGATGGAGATG
*Smpd1*  Forward   TGGGACTCCTTTGGATGGG
     Reverse   CGGCGCTATGGCACTGAAT
*Actin*   Forward  GGCTGTATTCCCCTCCATCG
     Reverse   CCAGTTGGTAACAATGCCATGT

### 4.17 RNA Sequencing (RNA‐Seq)

Total RNA was extracted using TRIzol reagent. Samples with an RNA Integrity Number (RIN) ≥ 7 were selected for downstream analysis. RNA transcriptome sequencing and subsequent bioinformatic analyses were performed by OE Biotechnology Co., Ltd. (Shanghai, China). Differentially expressed genes (DEGs) were identified using a threshold of p < 0.05 and a fold change > 1.2. Hierarchical clustering analysis was conducted to visualize gene expression patterns across experimental groups. Functional annotation of DEGs was performed through Gene Ontology (GO) enrichment analysis, Kyoto Encyclopedia of Genes and Genomes (KEGG) pathway analysis, and WikiPathways analysis to elucidate their biological significance.

### 4.18 Western Blotting

Protein expression was assessed by western blotting. Cell samples were lysed in 60 µL RIPA lysis buffer (KeyGEN BioTECH, China) containing protease inhibitors, phosphatase inhibitors, and phenylmethylsulfonyl fluoride (PMSF) for 30 min. Lysates were centrifuged at 13 000 g for 30 min at 4°C, and supernatants were collected as total protein. Protein concentration was determined using a BCA Protein Assay Kit (Beyotime, China). Equal amounts of protein (30 µg per sample) were separated by 10% SDS‐PAGE at 80 V for 30 min, followed by 120 V for 1 h. Proteins were transferred to 0.22 µm polyvinylidene fluoride (PVDF) membranes (Millipore) at 100 V for 100 min. Membranes were blocked with 5% skim milk for 2 h at room temperature and incubated overnight at 4°C with primary antibodies: AXL (R&D Systems, #AF854, 1:500), EGR1 (Proteintech, #22008‐1‐AP, 1:2500), *SMPD1* (Proteintech, #14609‐1‐AP, 1:800), 4‐HNE (Bioss, #bs‐6313r, 1:2000), and β‐Actin (Bioworld, #AP0060, 1:5000). After incubation with horseradish peroxidase (HRP)‐conjugated secondary antibodies for 2 h at room temperature, protein bands were visualized using an ECL chemiluminescence kit (Bioworld, USA) and imaged with a Gel‐Pro system (Tanon Technologies, China). Band intensity was quantified using ImageJ.

### 4.19 Chromatin Immunoprecipitation Quantitative PCR (ChIP‐qPCR)

ChIP‐qPCR was performed using the Hyperactive pG‐MNase CUT&RUN Assay Kit (Vazyme, China, HD101‐01) according to the manufacturer's instructions. Briefly, BV2 cells were treated with 5 µM EGR‐1‐IN‐1(MCE, HY‐163731) and the same volume of DMSO, then OGD for 3 h, reperfused and myelin stimulated for 6 h. 1 × 10^5^ living cells from each group were collected and bound to Concanavalin A‐coated Magnetic Beads Pro, and were incubated either anti‐EGR1 or control IgG antibodies to allow for specific binding overnight at 4°C. Then, the cell membrane was permeabilized using the non‐ionic detergent digitonin and the chromatin was incubated with pG‐MNase Enzyme with rotation at 4°C for 1 h. CaCl_2_ was added for 1.5 h on ice for fragmentation, and the reaction was terminated by adding stop buffer at 37°C for half an hour. The chromatin was eluted with TE buffer (1% SDS, 0.1 M NaHCO_3_), de‐crosslinked, and purified. qPCR was then performed using 2 × ChamQ Universal SYBR qPCR Master Mix. The primers designed based on EGR1 and *Smpd1* promoter binding Site‐1 and Site‐2 are as follows:

Forward GGCAGGTCAATCCGCTCAATGCA
Reverse CAGAACTGATGGAAGCCCTTTAGGGAAC

### 4.20 Enzyme‐Linked Immunosorbent Assay (ELISA)

Cell protein was harvested and diluted with PBS. Intracellular concentrations of mouse sphingomyelin (CB10565‐Mu, COIBO BIO, China) and *SMPD1* (RK08042, ABclonal, China) were quantified using commercial ELISA kits according to the manufacturer's protocols. The concentrations of proinflammatory cytokine in the Cre^+^ and Cre^−^ microglia culture supernatant of 6 h after OAM were quantified using commercial kits: Mouse TNF‐alpha ELISA Kit (RK00027, ABclonal, China), Mouse IL‐6 ELISA Kit (RK00008, ABclonal, China) and Mouse IL‐1 beta ELISA Kit (RK00006, ABclonal, China), strictly adhering to manufacturer's protocols. Optical density was measured using a Tecan microplate reader (Tecan, Switzerland) for accurate analyte quantification.

### 4.21 Statistical Analysis

Statistical analysis was performed using GraphPad Prism 8.0. Data were presented as mean ± SEM. For comparisons involving two independent groups, two‐tailed Student's *t*‐tests were used for normally distributed data, while the Mann‐Whitney *U* test was employed for non‐normal data (e.g., mNSS). Survival analysis was conducted using the Kaplan‐Meier method with Log‐rank test. For multiple group comparisons, one‐way ANOVA with Bonferroni post hoc test was applied to data with one independent variable. For experimental designs with two independent variables, two‐way ANOVA with Šídák's multiple comparisons test was utilized. Statistical significance was defined as *p* < 0.05.

## Author Contributions

Y.X. and M.Z. designed the project. J.J. and Y.G. designed and performed most of the experiments. J.L. and L.L. performed western blot; H.M. and M.S. performed CHIP; L.Y. and R.M. performed immunofluorescence; X.C. performed flow cytometry. S.X. performed tMCAO models. X.B. isolated primary cells. R.L. performed MRI DTI. M.Z. and Y.X. contributed reagents and materials. J.J. and Y.G. wrote the manuscript. The author(s) read and approved the final manuscript.

## Ethical Approval Statement

The animal study was conducted in accordance with the National Regulations of Experimental Animal Administration. All animal experimental protocols were approved by the Animal Care and Use Committee of the Model Animal Research Center, Nanjing Drum Tower Hospital, Affiliated Hospital of Medical School, Nanjing University (approval number:2025AE01036).

## Conflicts of Interest

The authors declare no conflicts of interest.

## Supporting information




**Supporting File**: advs73766‐sup‐0001‐SuppMat.docx.

**Supporting File**: advs73766‐sup‐0002‐Supp‐WB‐raw‐data.pdf.

## Data Availability

Raw and processed RNA‐seq data are deposited. Original data and uncropped western blotting images used to create all graphs are supplied. Any additional information required to reanalyze the data reported in this paper is available from the lead contact upon reasonable request.
