# Supplementary material for: AXL Promotes Ischemic Myelin Repair Through Alleviating Myelin Debris Deposition and Lipid Droplets Accumulation
Source: Adv Sci (Weinh). 2026 Jan 12;13(10):e17825. doi: 10.1002/advs.202517825 (PMC12915076; doi:10.1002/advs.202517825)
Supplement: Supplementary file 1 — Supporting File: advs73766‐sup‐0001‐SuppMat.docx. [file ADVS-13-e17825-s002.docx]

**Supporting Information**

**
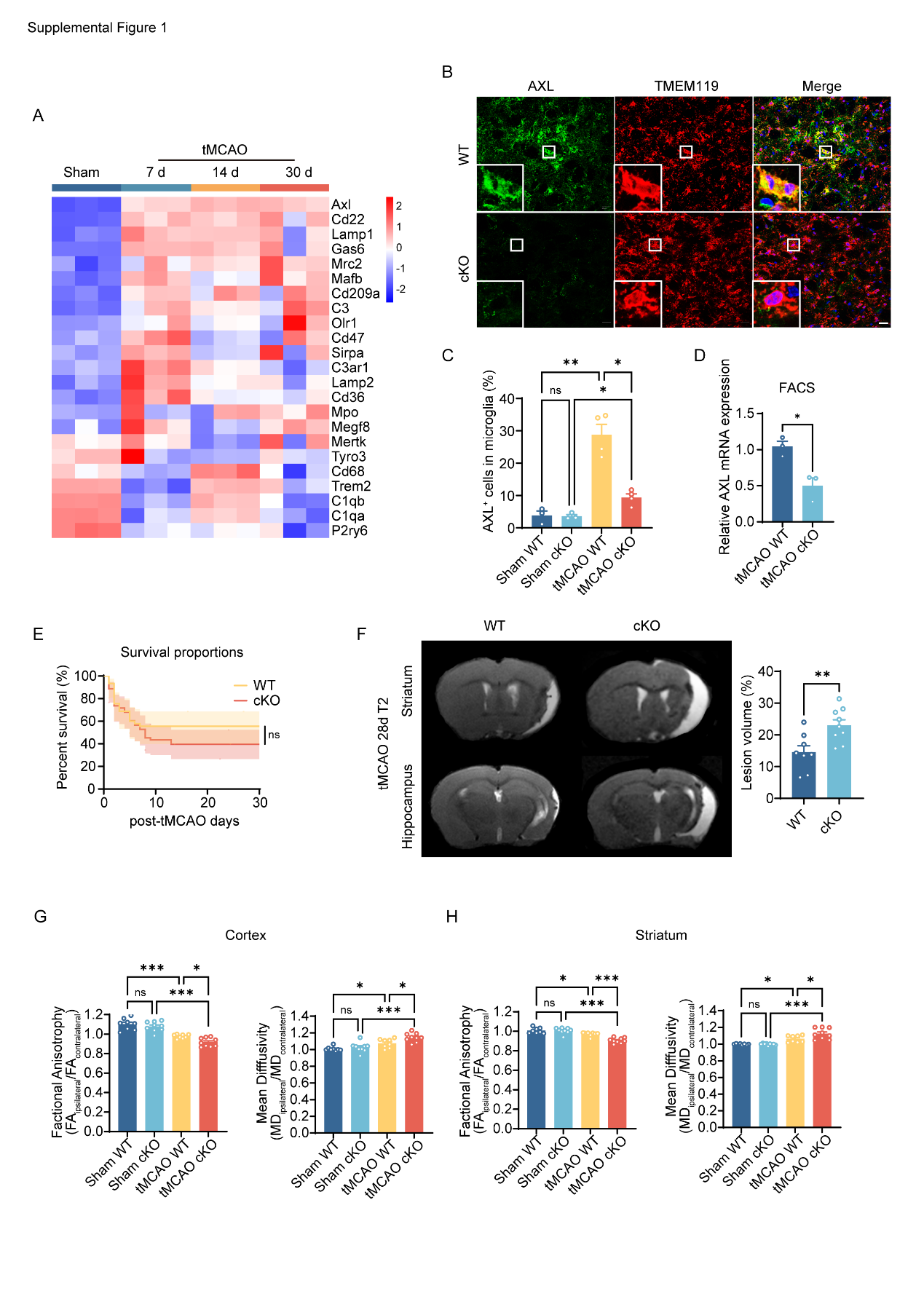
**

**Figure S1. Knockout efficiency of AXL cKO mice and the potential effect in tMCAO model.**

(A) Heat map of phagocytosis-related genes from sorting microglial transcriptomes at different time points after tMCAO.

(B) Representative images of AXL immunofluorescence staining of microglia in WT and AXL cKO mice at day 7 after tMCAO. Scale bar, 20 μm.

(C) Flow cytometry analysis of the percentage of AXL-positive microglia in WT and AXL cKO mice at day 7 after tMCAO. n ≥ 3 per group.

(D) QPCR detection of AXL mRNA in microglia of WT and AXL cKO mice by FACS at day 7 after tMCAO. n = 3 per group.

(E) Survival curves (95% CI) of WT and AXL cKO mice after tMCAO surgery.

(F) Representative T2WI and the analysis of infarct volume of WT and AXL cKO mice on day 28 post-tMCAO. n ≥ 8 per group.

(G, H) Quantification of FA ratio (ipsilateral/contralateral) and MD ratio (ipsilateral/contralateral) in cortex (G) and striatum area (H). n ≥ 8 per group.

Values were mean ± SEM. **p* < 0.05, ***p* < 0.01, ****p* < 0.001, and ns not significant.

**
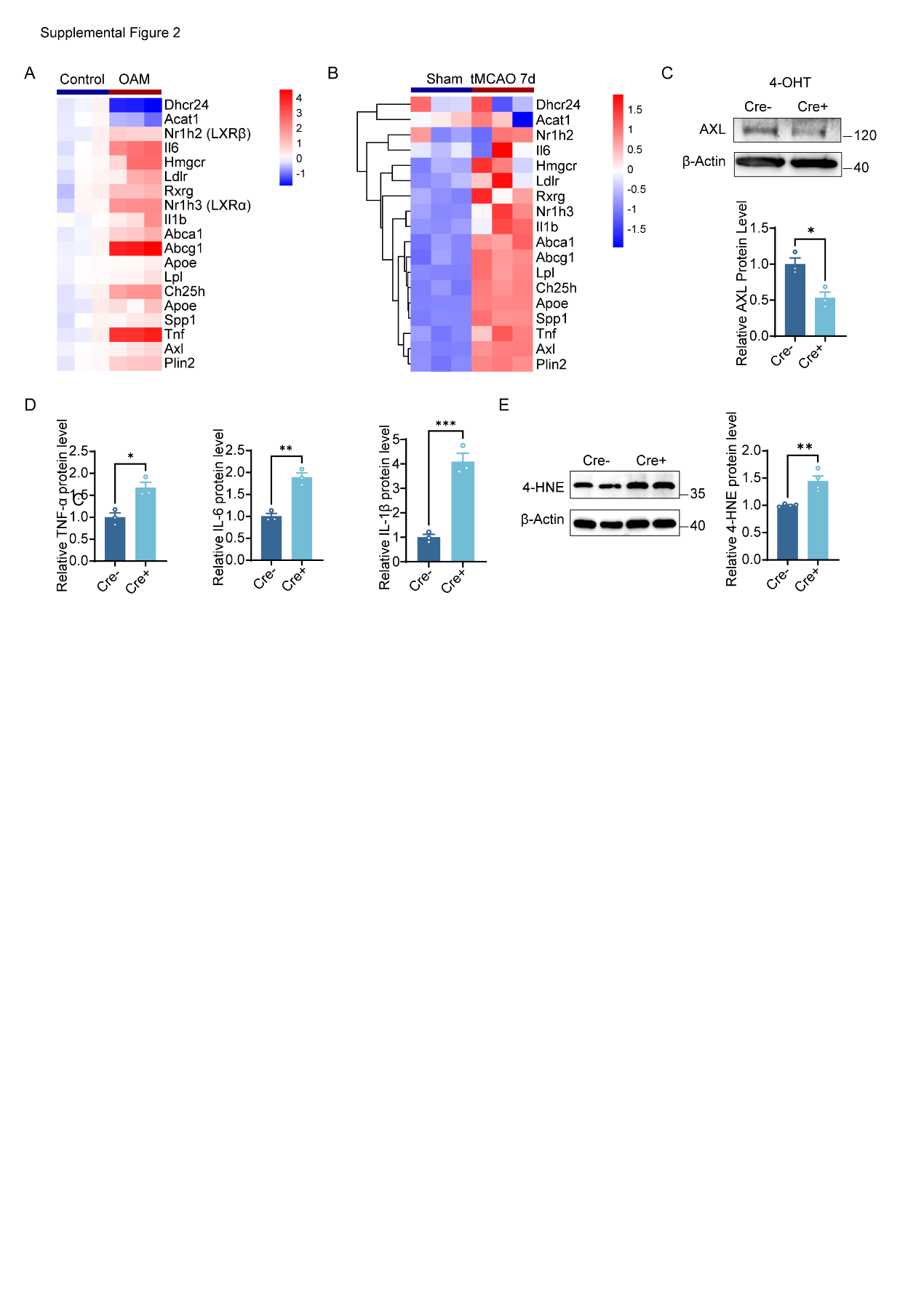
Figure S2. Axl deficient microglia showed dysregulated lipid metabolic genes, elevated inflammatory factors and accumulation of lipid peroxides**

(A) Heatmap of lipid metabolism-related genes expression of microglia after OAM *in vitro*. n = 3 per group.

(B) Heatmap of lipid metabolism-related genes expression of sham and tMCAO 7d sorting microglial transcriptomes *in vivo*. n = 3 per group.

(C) Western blot analysis of AXL in primary microglia extracted from AXL cKO mice pups and littermate control mice pups and treated with 4-OHT. 4-OHT: 4-hydroxy-tamoxifen. n = 3 per group.

(D) TNF-α, IL-6, and IL-1β protein levels were evaluated by ELISA in Cre^+^ and Cre^−^ microglia after OAM for 6 hours. n = 3 per group.

(E) 4-HNE protein level of Cre^+^ and Cre^−^ microglia after OAM for 6 hours. 4-HNE: 4-hydroxynonenal. n = 4 per group.

Values were mean ± SEM. **p* < 0.05, ***p* < 0.01, and ****p* < 0.001.

**
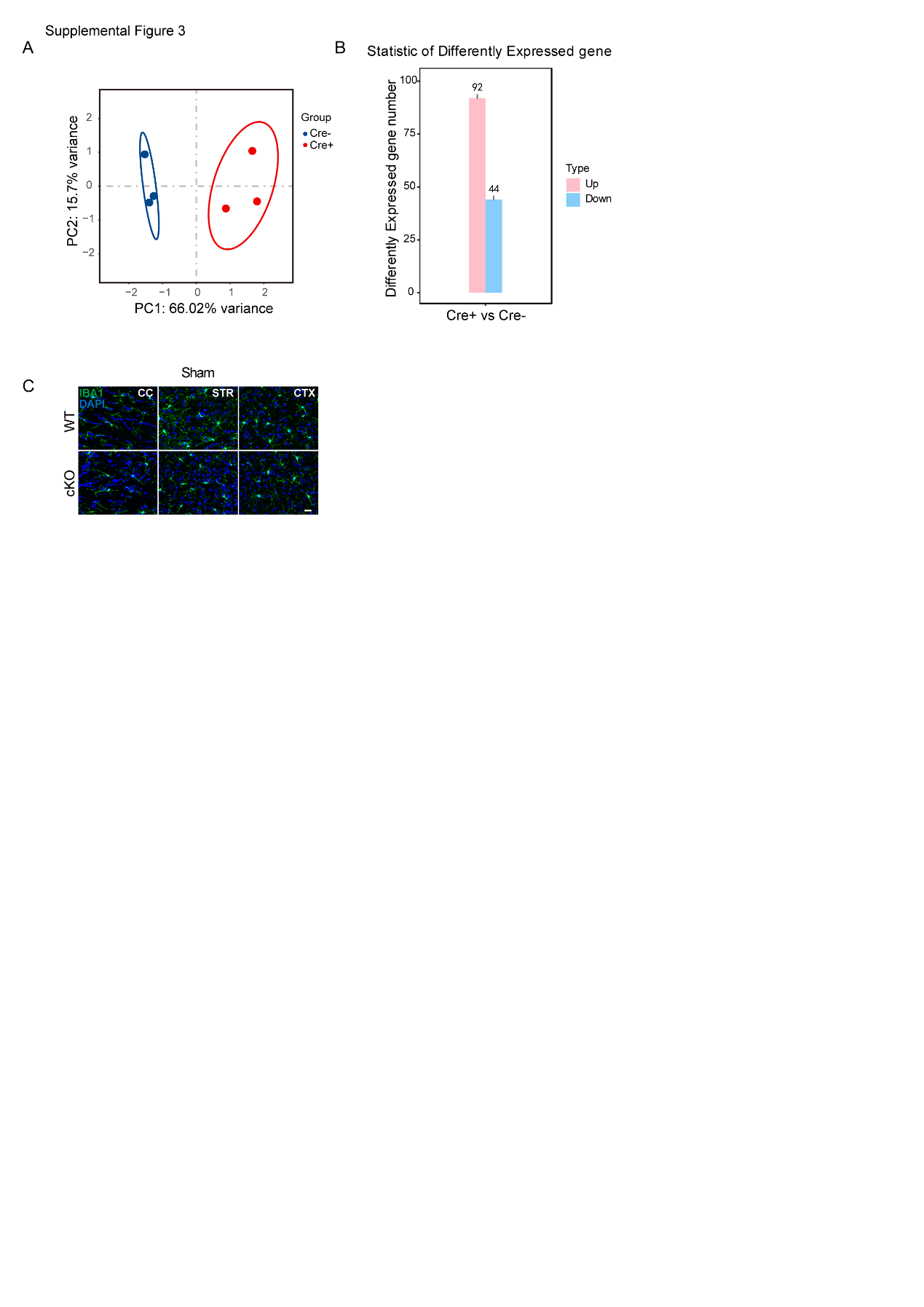
Figure S3. Transcriptomic analysis of Cre^+^ and Cre^−^ microglia and microglia characterization of intact WT and cKO mice**

(A) Principal component analysis (PCA) differentiating Cre^+^ microglia after OAM for 6 hours compared to Cre^−^ microglia. n = 3 per group.

(B) The significantly differentially expressed genes (DEGs) were shown between the Cre^+^ and Cre^−^ groups, including 92 up-regulated genes and 44 down-regulated genes (p < 0.05 and fold change > 1.2).

(C) Representative immunofluorescence images of microglia in WT and cKO mice in the sham group in CC (corpus callosum), STR (striatum), and CTX (cortex). Scale bar, 20 μm.

**
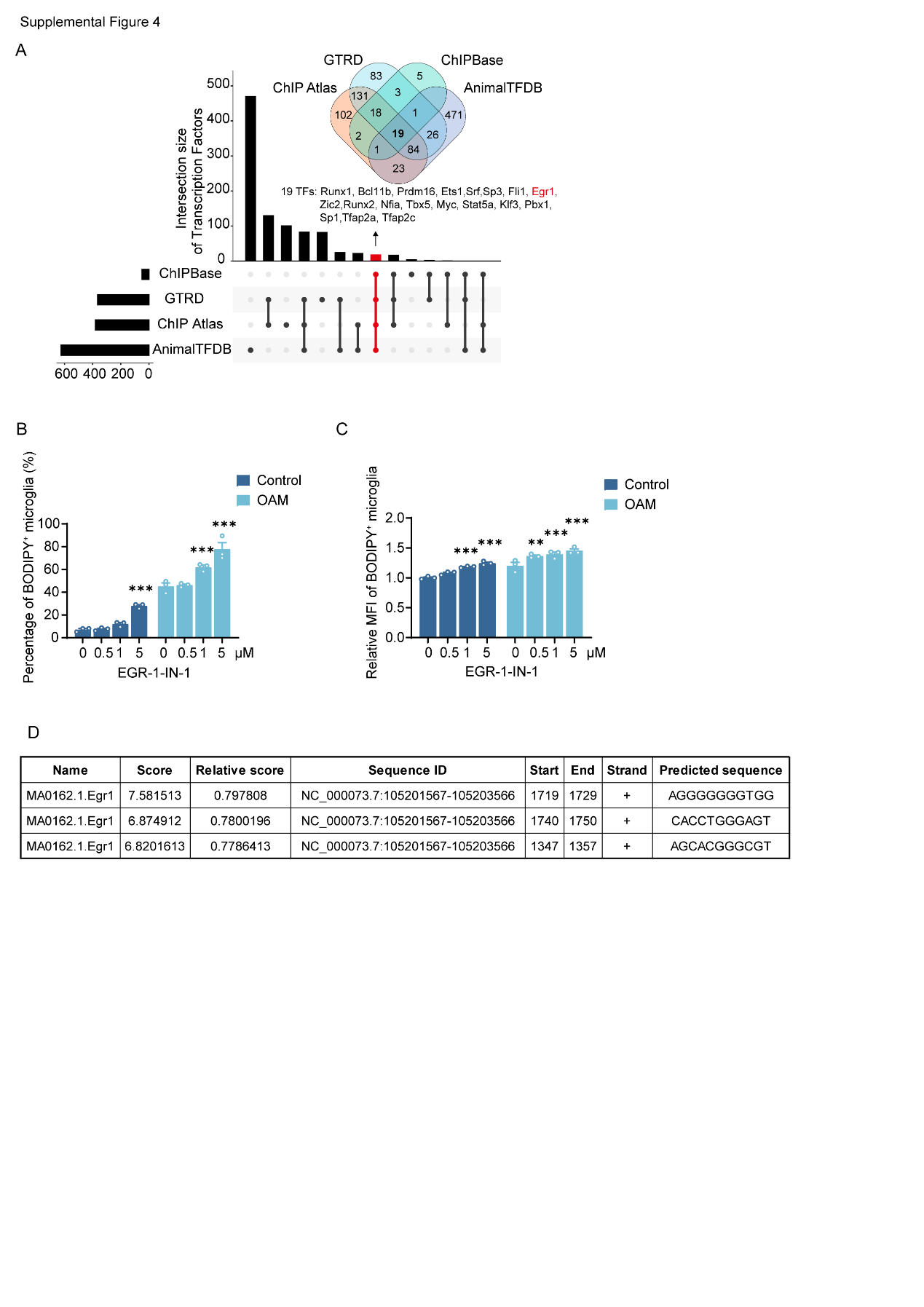
Figure S4. EGR1 is a transcription factor for *Smpd1*.**

(A) The intersection of SMPD1 transcription factors predicted by CHIPBase, GTRD, ChIP Atlas, and AnimalTFDB.

(B, C) The BODIPY^+^ microglia (B) and the MFI (C) after EGR-1-IN-1 treatment at 0, 0.5, 1, and 5μM. n = 3 per group. Values were mean ± SEM. ***p* < 0.01, and ****p* < 0.001.

(D) Detailed information on Top 3 binding sites of EGR1 in the promoter sequence of *Smpd1*.

**
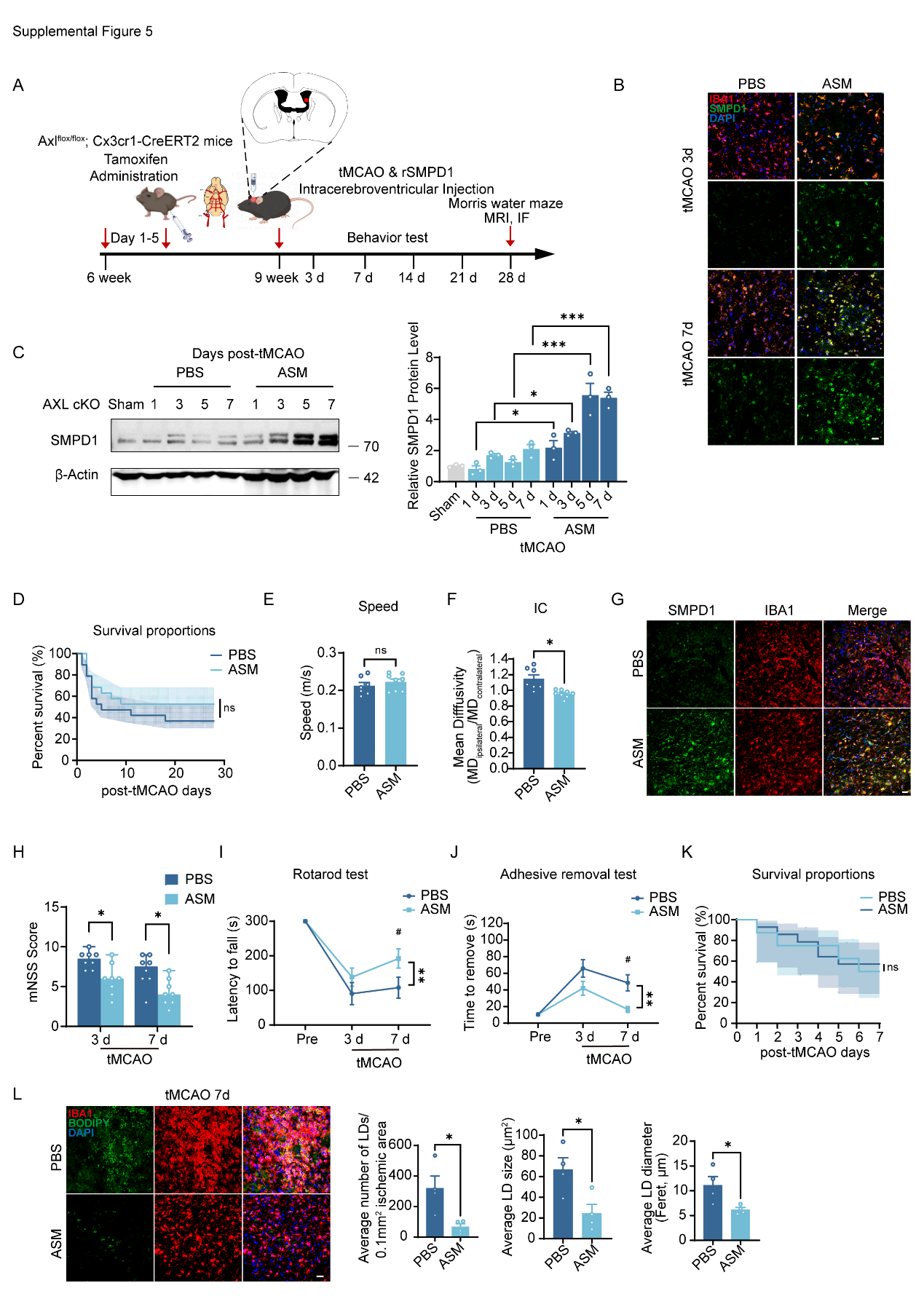
Figure S5. ASM supplementation alleviates ischemic white matter damage in AXL cKO mice.**

(A) Overview of the experimental timeline. Tamoxifen administration was initiated 3 weeks before tMCAO surgery. Recombinant ASM (rSMPD1) was administered intracerebroventricularly by intracranial stereotaxic injection immediately after tMCAO surgery. Behavioral tests were conducted 3, 7, 14, 21, and 28 days after surgery. and flow cytometry Morris water maze was performed 23-28 days after surgery. MRI and immunofluorescence staining were performed 28 days after surgery.

(B) Representative immunofluorescence images of ASM in the microglia of AXL cKO mice after PBS and ASM treatment at 3d and 7d post-tMCAO. Scale bar, 20 μm.

(C) Western blotting showed that after ASM administration, the SMPD1 protein level increased until 7th day post-tMCAO. n = 3 per group.

(D) Survival curves (95% CI) of AXL cKO mice treated with PBS and ASM administration after tMCAO surgery.

(E) The speed of PBS and ASM group evaluated by Morris water maze test. n = 7 for PBS group and n = 8 for ASM group.

(F) Quantification of MD value of PBS and ASM group expressed as the ratio of ipsilateral values to the contralateral values in the IC area. n = 7 for PBS group and n = 8 for ASM group.

(G) Representative images of SMPD1 and IBA1 immunofluorescence staining of AXL cKO mice treated with PBS and ASM 28 days after tMCAO. Scale bar, 20 μm.

(H-J) Modified Neurological Severity Scores (mNSS) (H), rotarod test (I), and adhesive removal test (J) in AXL cKO mice received stereotaxic injection of PBS or ASM at 24 hours post-tMCAO. n = 8 per group.

(K) Survival curves (95% CI) of AXL cKO mice treated with PBS and ASM administration at 24 hours post-tMCAO.

(L) Representative immunofluorescence images of BODIPY staining, number of LDs, LD size, and LD diameter in the ipsilateral striatum at 7d post-tMCAO of AXL cKO mice which received PBS and ASM treatment 24h post-tMCAO. Scale bar, 20 μm. n = 4 per group.

Modified Neurological Severity Scores (A) are shown as median ± ranges, and other values are presented as mean ± SEM. **p* < 0.05, ***p* < 0.01, ****p* < 0.001, and ns not significant. ^#^*p* < 0.05 compared between PBS and ASM group on each day.
