# Supplementary material for: AXL Promotes Ischemic Myelin Repair Through Alleviating Myelin Debris Deposition and Lipid Droplets Accumulation
Source: Adv Sci (Weinh). 2026 Jan 12;13(10):e17825. doi: 10.1002/advs.202517825 (PMC12915076; doi:10.1002/advs.202517825)

Figure 7C

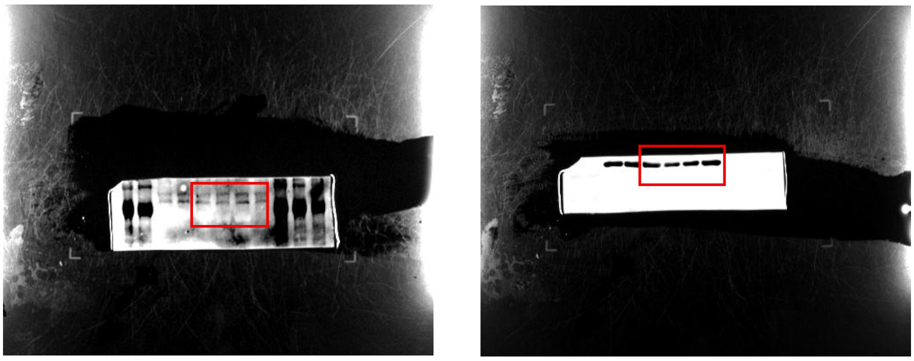

Figure 7F

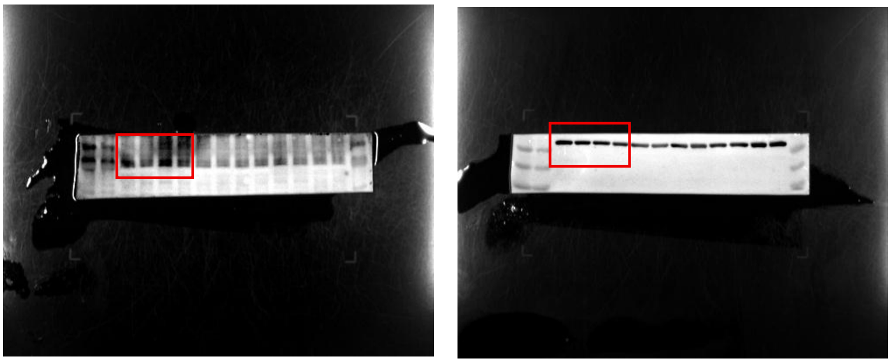

Figure 7G

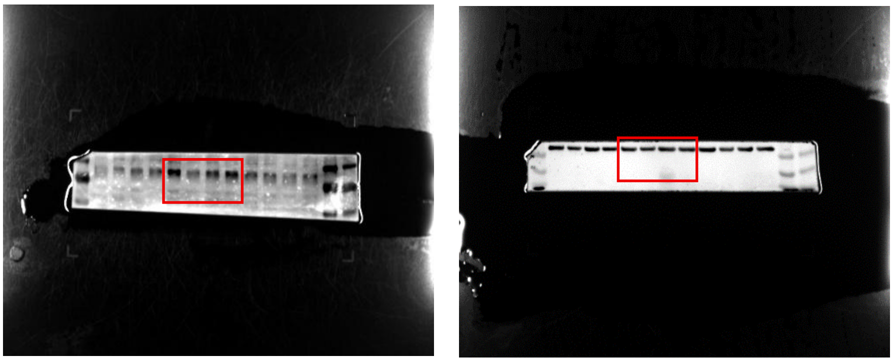

Figure S2C, Supporting Information

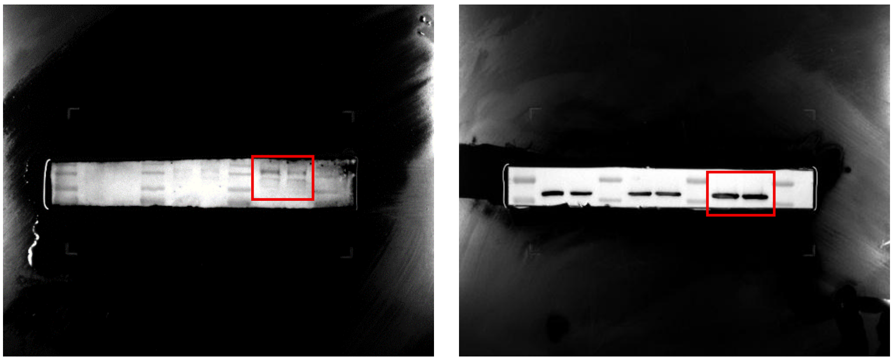

Figure S2E, Supporting Information

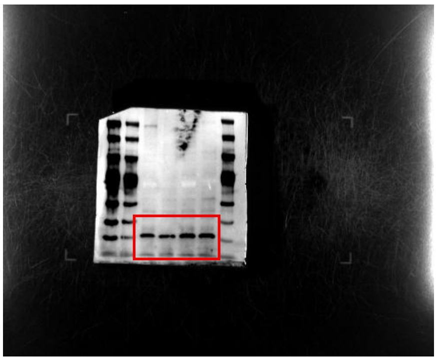

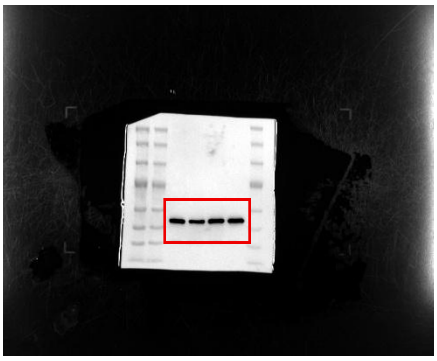

Figure S5C, Supporting Information

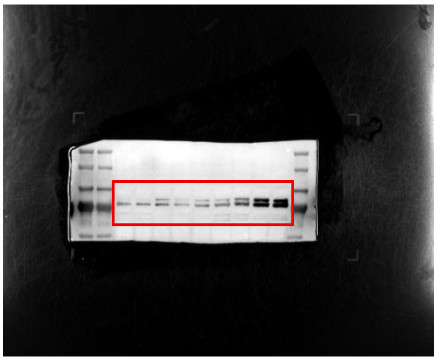

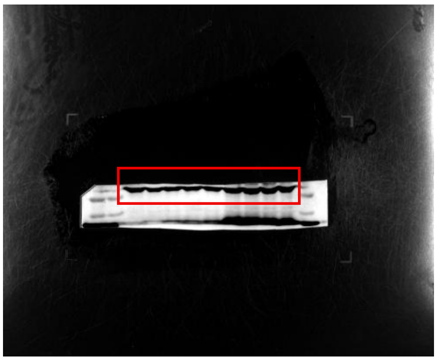

Supplement: Supplementary file 2 — Supporting File: advs73766‐sup‐0002‐Supp‐WB‐raw‐data.pdf. [file ADVS-13-e17825-s001.pdf]
